# Loss of retinoid X receptor gamma subunit impairs group 1 mGluR mediated electrophysiological responses and group 1 mGluR dependent behaviors

**DOI:** 10.1038/s41598-021-84943-x

**Published:** 2021-03-10

**Authors:** Chirag Upreti, Caitlin M. Woodruff, Xiao-Lei Zhang, Michael J. Yim, Zhen-yu Zhou, Andrew M. Pagano, Dina S. Rehanian, Deqi Yin, Eric R. Kandel, Patric K. Stanton, Russell E. Nicholls

**Affiliations:** 1 Department of Cell Biology and Anatomy, New York Medical College, Valhalla, NY 10595 USA; 2 Department of Neurology, New York Medical College, Valhalla, NY 10595 USA; 3 Department of Neuroscience, Columbia University, 3227 Broadway, New York, NY 10027 USA; 4 Department of Pathology and Cell Biology, Columbia University, 630 West 168thStreet, New York, NY 10032 USA; 5 Taub Institute for Research on Alzheimer’s Disease and Aging Brain, Columbia University, 630 West 168thStreet, New York, NY 10032 USA; 6 Howard Hughes Medical Institute, Columbia University, 3227 Broadway, New York, NY 10027 USA; 7 Kavli Institute for Brain Science, Columbia University, 3227 Broadway, New York, NY 10027 USA; 8 Mortimer B. Zuckerman Mind Brain Behavior Institute, Columbia University, 3227 Broadway, New York, NY 10027 USA

**Keywords:** Molecular neuroscience, Neuronal physiology, Synaptic plasticity

## Abstract

Retinoid X receptors are members of the nuclear receptor family that regulate gene expression in response to retinoic acid and related ligands. Group 1 metabotropic glutamate receptors are G-protein coupled transmembrane receptors that activate intracellular signaling cascades in response to the neurotransmitter, glutamate. These two classes of molecules have been studied independently and found to play important roles in regulating neuronal physiology with potential clinical implications for disorders such as depression, schizophrenia, Parkinson’s and Alzheimer’s disease. Here we show that mice lacking the retinoid X receptor subunit, RXRγ, exhibit impairments in group 1 mGluR-mediated electrophysiological responses at hippocampal Schaffer collateral-CA1 pyramidal cell synapses, including impaired group 1 mGluR-dependent long-term synaptic depression (LTD), reduced group 1 mGluR-induced calcium release, and loss of group 1 mGluR-activated voltage-sensitive currents. These animals also exhibit impairments in a subset of group 1 mGluR-dependent behaviors, including motor performance, spatial object recognition, and prepulse inhibition. Together, these observations demonstrate convergence between the RXRγ and group 1 mGluR signaling pathways that may function to coordinate their regulation of neuronal activity. They also identify RXRγ as a potential target for the treatment of disorders in which group 1 mGluR signaling has been implicated.

## Introduction

Proper control of neuronal activity and synaptic transmission is critical for normal nervous system function, and accordingly, multiple neurotransmitters, neuromodulators, and signaling pathways have been found to participate in their regulation. However, neurons do not respond to these factors in isolation. To produce appropriate responses, neurons need to integrate multiple inputs.

Here we identify an interaction between two pathways known to play important roles in regulating neuronal and synaptic activity: (1) group 1 metabotropic glutamate receptors (mGluRs) and (2) the nuclear hormone receptor family member, retinoid X receptor gamma (RXRγ). Specifically, we found that animals lacking RXRγ exhibited impairments in group 1 mGluR-mediated synaptic transmission and synaptic plasticity, together with impairments in group 1 mGluR-mediated behaviors. These data suggest an interaction between these pathways in normal learning and memory and in the control of disease-relevant behaviors including Parkinson’s disease-related motor impairments, and Schizophrenia-related executive function.

RXRγ is one of three retinoid X receptors (α, β, γ) found in mice and humans with partially overlapping expression patterns and functions. RXRs are members of the nuclear receptor superfamily and form heterodimers with other nuclear receptor family members that bind to discrete DNA sequences to regulate transcription in response to ligand binding (reviewed in^1–3^). RXR heterterodimers can be either permissive with respect to ligand-dependent transactivation (activated by either RXR ligand or heterodimer partner ligands), or they can be non-permissive (activated by heterodimer partner ligands only). RXR homodimers also bind to DNA and regulate transcription in a ligand-dependent manner. However, the identity of endogenous RXR ligands remains an area of active investigation^4^. In addition to their roles as transcription factors, RXRs have been found to act through non-genomic mechanisms to regulate apoptosis^5^, platelet activation^6,7^, and phosphoinositide 3-kinase(PI3K)/Akt signaling^8^. Currently, RXR agonists are used in the treatment of cutaneous T-cell lymphoma, breast and lung cancers^3,9^ and are being pursued for use in treating Alzheimer’s disease^10–15^, Parkinson’s disease^16^ and schizophrenia^17^. Previous studies of mice lacking RXRγ have implicated these receptors in activity-dependent, long-term synaptic plasticity^18^, in remyelinization^19^, as well as in behaviors related to depression^20,21^, schizophrenia^21,22^, and Parkinson’s disease-related motor impairments^23^.

The metabotropic glutamate receptors are G-protein coupled transmembrane receptors for the neurotransmitter glutamate that are responsible for initiating intracellular signaling cascades leading to both transient and persistent changes in neuronal excitability and synaptic plasticity. The mGluRs are divided into three groups based on sequence similarity and G-protein coupling. Group 1 consists of 2 members—mGlur1 and mGlur5—both of which are coupled to Gα_q/ll_-containing heterotrimeric G-proteins. Canonically, G_q/11_ activation leads to activation of phospholipase C, and the production of inositol 1,4,5-trisphosphate (IP3) and diacylglycerol. This in turn leads to activation of protein kinase C (PKC) and the release of calcium from intracellular stores^24^. In addition, multiple additional downstream effectors of group 1 mGluR activation have been identified, including phospholipases D and A2, c-Jun N-terminal kinase (JNK), mitogen-activated protein kinase/extracellular receptor kinase (MAPK/ERK), and the mammalian target of rapamycin (mTOR) (see^24–26^ for recent reviews). A host of other proteins have also been found to interact with group 1 mGluRs to regulate or mediate their signaling functions. The most well-characterized of these proteins are the postsynaptic scaffolding Homer proteins that link group 1 mGluRs to other postsynaptic proteins, including the NMDA receptor^27^. Additional, functionally important interactions have also been described between group 1 mGluRs and various downstream effectors including caveolin, norbin, prolyl-isomerase 1 (Pin1), cellular prion protein (PrP^c^), optineurin, and Preso1 (reviewed in^28^). Through their various effectors, group 1 mGluRs regulate critical neuronal functions, including NMDA receptor activity, release of internal calcium, protein translation, and production of endocannabinoids. At the synaptic level, group 1 mGluRs are required for normal homeostatic scaling of synaptic activity^29,30^, for various forms of long-term synaptic potentiation (LTP), and for multiple forms of long-term synaptic depression (LTD) that, depending on synapse type, are produced via pre- or postsynaptic, protein synthesis dependent or independent, mechanisms^31,32^. Given this diversity of downstream effects, it is therefore, not surprising that group 1 mGluRs have been implicated in a number of neurological and neuropsychiatric disorders including schizophrenia, depression, anxiety, autism, addiction, Alzheimer’s disease, Huntington’s disease, Parkinson’s disease, and fragile X syndrome (reviewed in^24^).

In earlier studies, RXRγ knockout mice were found to exhibit impaired stimulus-evoked LTD, but normal LTP and depotentiation^18^. The data we present here show that these animals also exhibit impairments in multiple group 1 mGluR-dependent electrophysiological responses, including group 1 mGluR-dependent LTD, group 1 mGluR-dependent calcium release, and group 1 mGluR-activated voltage sensitive currents. Moreover, our behavioral characterization of these animals revealed impairments in a subset of group 1 mGluR-dependent behaviors including motor performance, spatial object recognition, and prepulse inhibition. Together our data suggest that RXRγ is required for normal group 1 mGluR-dependent modulation of synaptic transmission and plasticity, and that these two pathways interact on the molecular level to regulate normal and disease-related nervous system function.

## Results

### RXRγ is required for group 1 mGluR-dependent long-term depression (LTD) of synaptic strength, Ca^2+^ release from intracellular stores and voltage-dependent cation currents

**Figure 1:**
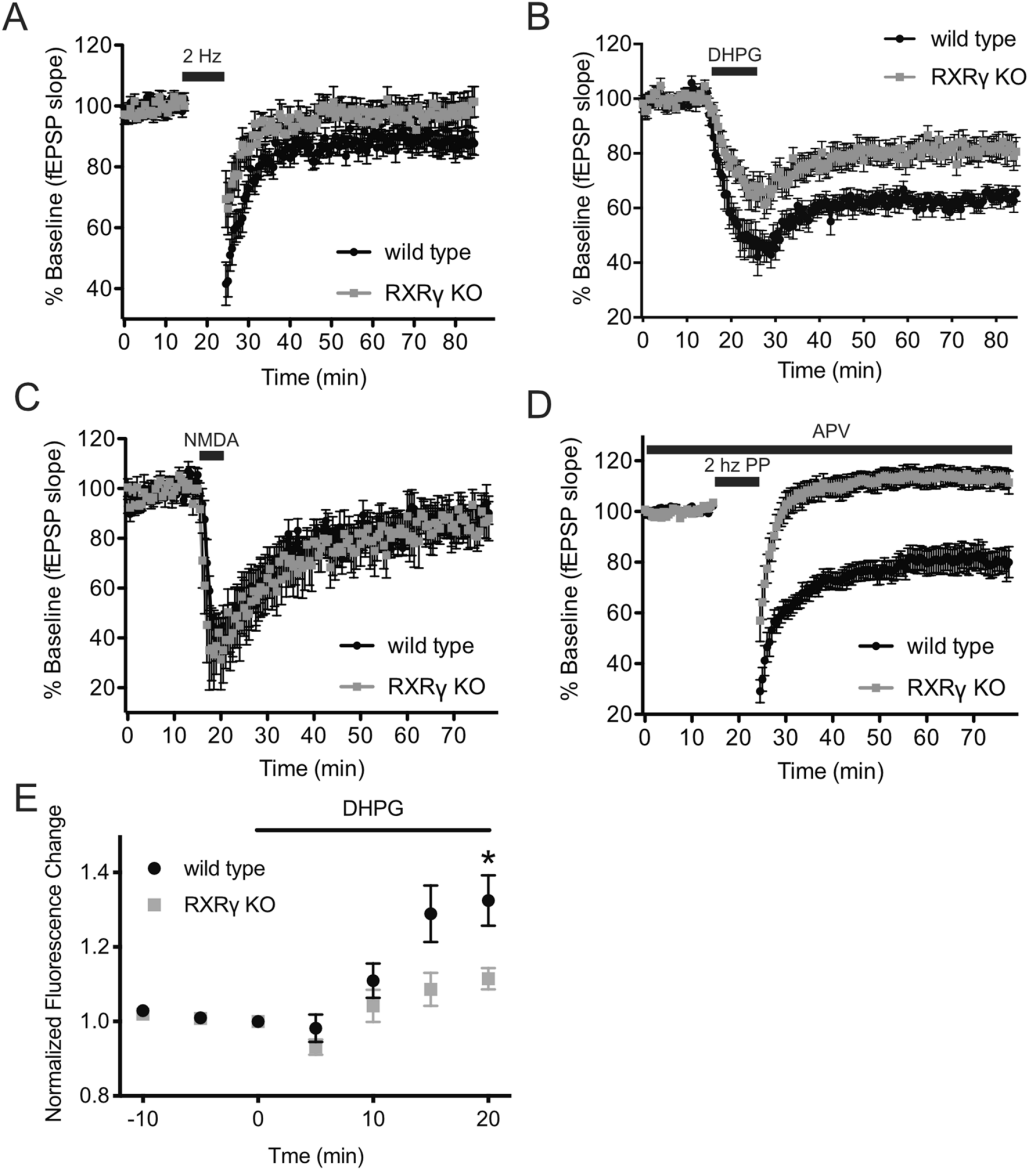
RXRγ KO mice exhibit deficits in group 1 mGluR-dependent LTD and impaired group 1 mGluR-activated calcium release. (**A**–**D**) show time courses of averaged initial fEPSP slopes ± SEM at Schaffer collateral-CA1 synapses in hippocampal slices from wild-type (WT) vs. RXRγ knockout (KO) mice. In (**A**–**D**), mean responses relative to baseline were calculated for each slice over 50–55 min post-stimulation/application and compared between groups using Student’s t-tests. (**A**) LTD at Schaffer collateral-CA1 synapses evoked by 2 Hz/10 min low frequency stimulation (black bar) was eliminated in hippocampal slices from RXRγ-KO mice (98.8 ± 4.6% of baseline, grey circles, N = 11 slices from 7 animals) compared to slices from wild-type animals (87.9 ± 3.7% of baseline, black circles, N = 14 slices from 7 animals) (t = 2.1, P = 0.045). (**B**) LTD evoked by 10 min bath application of 30 μM DHPG (solid bar) was significantly smaller in hippocampal slices from RXRγ-KO mice (82 ± 3.9% of baseline, grey circles, N = 15 slices from 10 animals) compared to wild-type mice (63 ± 2.6% of baseline, black circles, N = 12 slices from 5 animals) (t = 3.605, P = 0.0014). (**C**) LTD evoked by 5 min bath application of 20 μM NMDA (solid bar) was not significantly different in hippocampal slices from RXRγ-knockout mice (86 ± 5.3% of baseline, grey circles, N = 8 slices from 7 animals) compared to slices from wild-type mice (89 ± 3.9% of baseline, black circles, N = 7 slices from 7 animals) (t = 1.951, P = 0.0652). (**D**) LTD evoked by 2 Hz/10 min train of pairs of stimuli (interpulse interval 50 ms) in the presence of the NMDA receptor antagonist D-AP5 (10 µM) to isolate mGluR-dependent LTD, was converted to modest potentiation in hippocampal slices from RXRγ KO mice (113 ± 6.2% of baseline grey circles, N = 11 slices from 6 animals), compared to wild type mice (79 ± 4.4% of baseline black circles, N = 11 slices from 5 animals) (t = 87.11, P < 0.0001). (**E**) DHPG-induced Ca++ release measured using the calcium sensitive dye Calcium Green and two-photon laser scanning microscopy imaging was significantly smaller in RXRγ KO CA1 pyramidal neurons, compared to wild-type control neurons (WT 32.4 ± 7% increase, KO 11.5 ± 2.5% increase, P = 0.029, Student’s t-test, t = 2.841, N = 4 cells per group). DHPG was bath applied to slices at 30 μM for 20 min beginning at time = 0. Mean ± SEM Calcium Green fluorescence intensity normalized to pretreatment baseline were calculated at 5, 10, 15 and 20 min of DHPG application. Data were plotted using Prism (https://www.graphpad.com/scientific-software/prism/) and the figure assembled using Affinity Designer (https://affinity.serif.com/en-gb/designer/) software.

RXRγ has been reported previously to be required for induction of stimulus-evoked LTD at hippocampal Schaffer collateral-CA1 synapses^18^. We reproduced this result using a 2 Hz/10 min stimulation protocol that elicited persistent LTD in slices prepared from wild-type animals, but significantly less LTD in slices from RXRγ knockout littermates (Fig. 1A). Since LTD can be induced at these synapses via at least two distinct mechanisms—one dependent on NMDA receptor activity and another dependent on group 1 mGluR activity^33,34^, we sought to determine whether RXRγ was required for one or both of these forms of LTD. We elicited mGluR-dependent or NMDA receptor-dependent LTD in acute slices from wild-type and RXRγ knockout mice by direct pharmacological activation of these receptors using the agonists DHPG or NMDA. Surprisingly—given the dependence of low-frequency dependent LTD on NMDA receptor activation^35,36^ (Fig. S1)—we found that RXRγ knockout significantly impaired LTD induced by application of the group 1 mGluR agonist, DHPG (Fig. 1B), but had no significant effect on LTD induced by application of NMDA (Fig. 1C). An NMDA receptor-independent form of LTD that depends on group 1 mGluR activation, but may also involve contribution of additional receptor types, can be produced by administration of low frequency paired-pulse stimulation in the presence of an NMDA receptor antagonist^37–40^ (Fig. S1). This protocol produced a robust LTD of evoked responses in slices from wild-type animals, but actually produced modestly *potentiated* responses in animals lacking RXRγ (Fig. 1D), further suggesting that RXRγ knockout impairs LTD elicited by group 1 mGluR activation.

Since these results suggested that RXRγ knockout interferes with LTD produced by group 1 mGluR activation, we sought to determine whether RXRγ might also be required for other group 1 mGluR-mediated electrophysiological responses. One such response is the release of Ca^2+^ from internal stores that results from group 1 mGluR activation. To visualize dendritic [Ca^2+^], we loaded CA1 pyramidal neurons in hippocampal slices with the calcium sensitive dye, Calcium Green-1 (100 µM), and measured calcium release in these cells using two-photon laser scanning microscopic imaging^41^. Bath application of the group 1 mGluR agonist, DHPG (30 μM), elicited a time-dependent change in mean fluorescence intensity that was significantly smaller in CA1 pyramidal neurons lacking RXRγ, compared to wild-type control neurons (Fig. 1E). These data suggest that, in addition to group 1 mGluR-dependent LTD, RXRγ is also required for normal group 1 mGluR-induced release of Ca^2+^ from intracellular stores.

**Figure 2:**
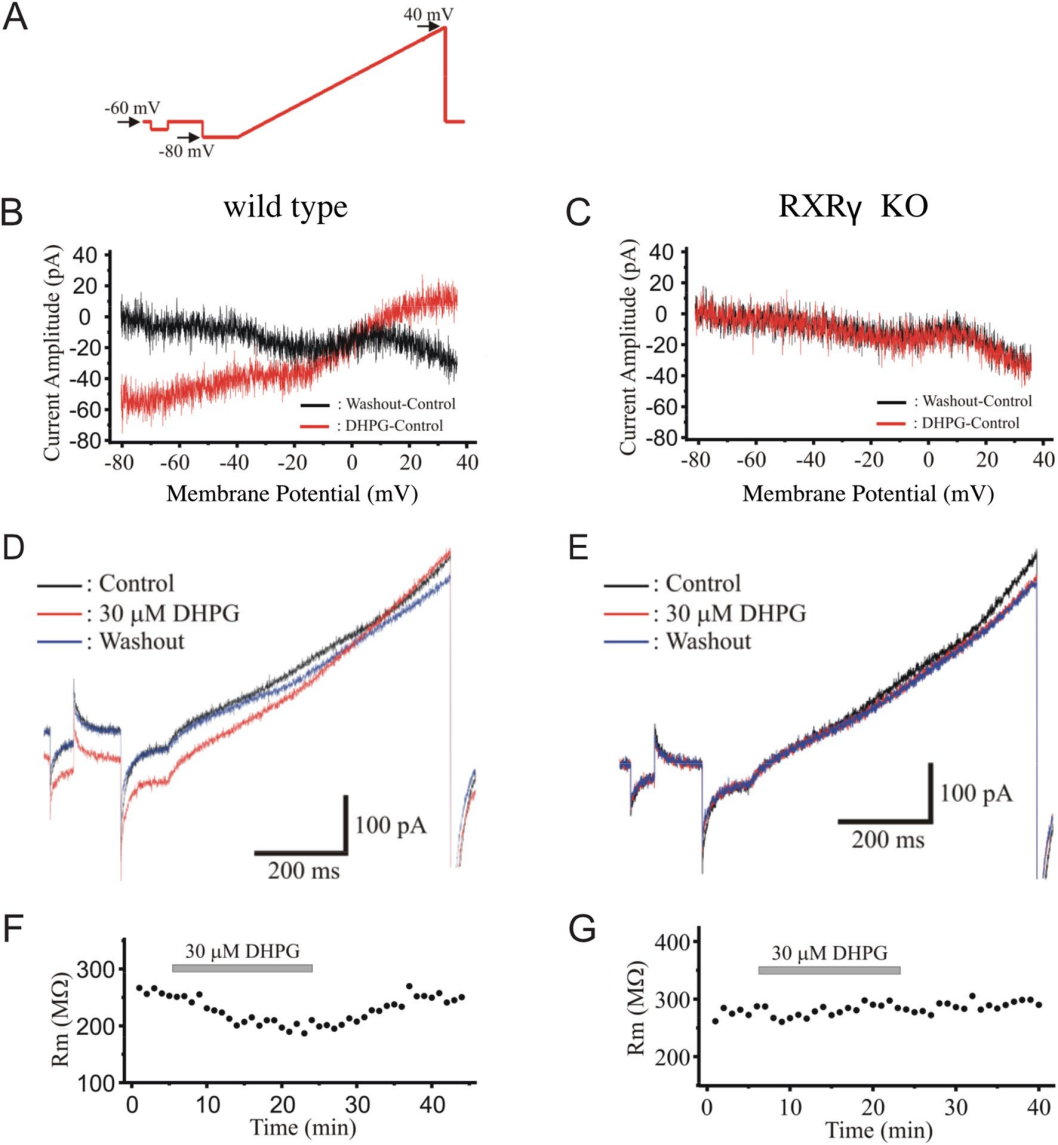
RXRγ KO mice exhibit impaired group 1 mGluR-activated voltage-sensitive currents. (**A**) Schematic of voltage-clamp stimulation ramp to elicit voltage-sensitive inward currents. (**B**) Sample current/voltage relations in a wild type CA1 pyramidal neuron during bath-application of the group I agonist DHPG (30 µM, red trace, subtraction of pre-drug baseline I/V plot from I/V relation after 20 min application of DHPG) compared to washout control (black trace) in the same cell. (**C**) Sample current/voltage relations in an RXRγ KO CA1 pyramidal neuron during bath-application of DHPG (30 µM, red trace, subtraction of pre-drug baseline I/V plot from I/V relation after 20 min application of DHPG) compared to washout control (black trace) in the same cell. (**D**) Sample inward currents in a wild type CA1 pyramidal neuron before (black trace), during (red trace), and after (blue trace) bath application of 30 µM DHPG (WT peak control current: 40.8 ± 11.4 pA; peak current in presence of DHPG: 21.7 ± 9.6 pA, N = 7 cells, (paired t-test; t = 2.788, P = 0.0317). (**E**) Sample inward currents in an RXRγ KO CA1 pyramidal neuron before (black trace), during (red trace), and after (blue trace) bath application of 30 µM DHPG (KO peak control current: 37.3 ± 16.5 pA; peak current in presence of DHPG: 42.5 ± 17.6 pA, N = -6 cells, (paired t-test; t = 0.552, P = 0.6044). (**F**) DHPG (30 µM, grey bar) reduced voltage-sensitive inward currents, plotted as reduction in membrane input resistance Rm, in wild type control neurons. (**G**) DHPG (30 µM, grey bar) did not alter voltage-sensitive inward currents in CA1 pyramidal neurons in slices from RXRγ KO mice. Data were plotted using Origin Pro (https://www.originlab.com) and the figure assembled using Affinity Designer (https://affinity.serif.com/en-gb/designer/) software.

A third group 1 mGluR-mediated response that we assessed was group 1 mGluR-mediated activation of voltage-sensitive inward current^42^. To do this, we performed whole-cell patch-clamp recordings from CA1 pyramidal neurons in hippocampal slices from wild-type animals and their RXRγ knockout siblings. We found that, in wild-type cells, bath application of the group 1 mGluR agonist DHPG (30 µM) elicited a voltage-dependent inward current, but that this current was absent in cells from RXRγ knockout animals (Fig. 2). This blockade of DHPG-induced voltage-sensitive currents is consistent with the effect of RXRγ knockout on mGluR-dependent LTD and mGluR agonist-evoked calcium release, further supporting the conclusion that a lack of RXRγ results in marked impairment in group 1 mGluR signaling in these animals.

### RXRγ does not affect group 1 mGluR expression

**Figure 3:**
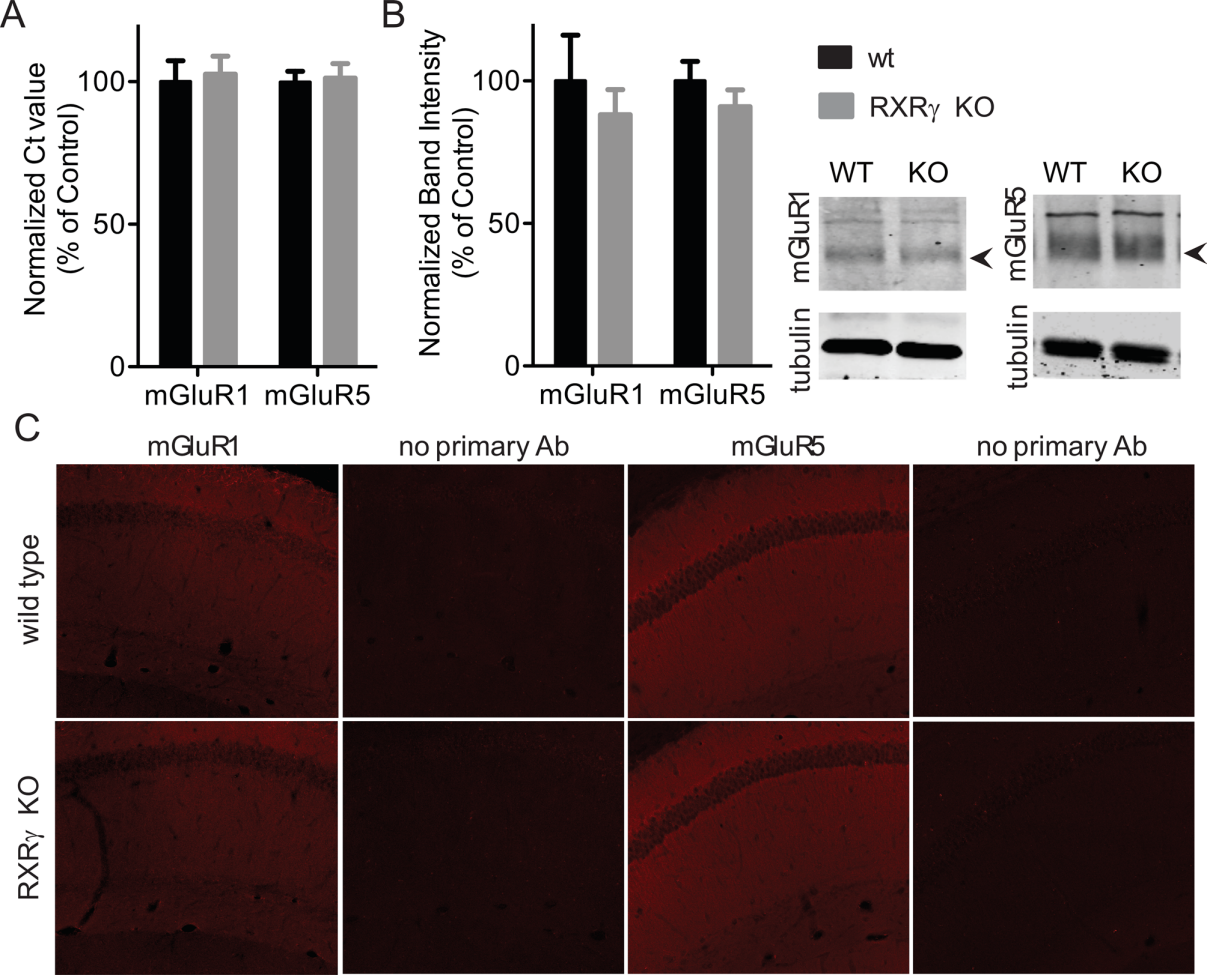
Loss of RXRγ does not affect mGluR1 or mGluR5 expression. (**A**) mGluR1 and mGluR5 RNA is present in equivalent amounts in hippocampal homogenates from RXRγ KO and WT mice as measured by quantitative RT-PCR (T-test: t = 0.2564, P = 0.8028 for mGluR1 and t = 0.2093, P = 0.8384 for mGluR5, N = 6 animals per group run in triplicate). (**B**) mGluR1 and mGluR5 protein is present in equivalent amounts in hippocampal homogenates from RXRγ KO and WT animals as measured by quantitative western blotting. N = 4 animals per group run in duplicate*.* No significant differences between genotypes for either protein by T-test (t = 0.6477, P = 0.5412 for mGluR1 and t = 1.008, P = 0.3217 for mGluR5). At right, representative images of western blots showing anti-mGluR1 or mGluR5 and corresponding anti-tubulin immunoreactivity from WT and RXRγ KO mice. (See also uncropped images in Fig. S1). (**C**) Comparable levels and distribution of mGluR1 and mGluR5 protein in the hippocampal CA1 region by qualitative immunohistochemistry. Representative images of immunostained hippocampal CA1 region tissue in sections prepared from 3 animals per genotype processed in parallel with anti-mGluR1 or anti-mGluR5 primary antibody or with primary antibody omitted. Data were plotted using Prism (https://www.graphpad.com/scientific-software/prism/) and images prepared using Image Studio (https://www.licor.com/bio/image-studio/) and Olympus Fluoview (http://www.olympusconfocal.com/products/fv1000/fv1000software.html) software. The figure was assembled using Affinity Designer (https://affinity.serif.com/en-gb/designer/) software.

One of the ways in which loss of RXRγ could result in reduced group 1 mGluR signaling is through reduced group 1 mGluR expression. To test this possibility, we compared mGluR1 and mGlur5 expression in hippocampal homogenates and slices from wild-type and RXRγ knockout animals. Quantitative RT-PCR using mGluR1 or mGluR5 specific primers revealed no significant differences in RNA expression levels for these two genes between wild-types and knockouts (Fig. 3A), suggesting that loss of RXRγ does not affect group 1 mGluR signaling through reductions in mGluR1 or mGluR5 mRNA expression. To test for possible differences in mGluR1 or mGluR5 protein levels, we also performed quantitative western blot analysis on hippocampal homogenates from wild-type and RXRγ knockout animals and found comparable levels of these proteins in both groups (Fig. 3B). Finally, to test for possible qualitative differences in the distribution of these two receptors, we performed immunohistochemistry on hippocampal sections from these animals, and found no gross differences in mGluR1 or mGluR5 distribution when we compared wild-type and RXRγ knockout mice (Fig. 3C). Together, these data suggest that, while loss of RXRγ impairs group 1 mGluR signaling, this is not due to reduced mGluR1 or mGluR5 expression or gross alterations in group 1 mGluR distribution in these animals.

Since our data suggested that loss of RXRγ impairs group 1 mGluR-induced electrophysiological responses, we next sought to determine the extent to which loss of RXRγ might also affect disease-relevant behavioral processes known to involve group 1 mGluR activity.

### RXRγ knockout does not alter anxiety in a novel open field environment or elevated plus maze

**Figure 4:**
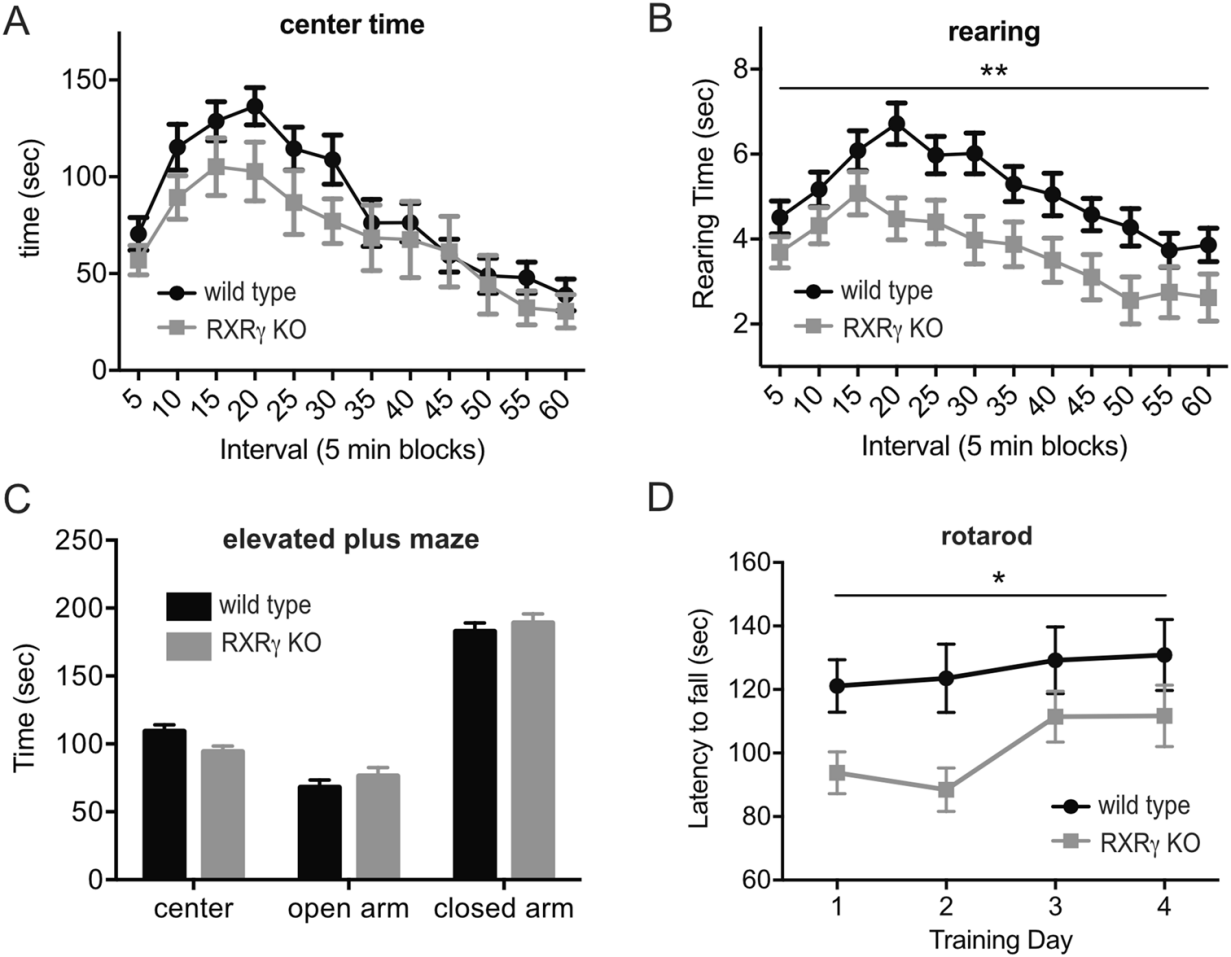
Animals lacking RXRγ exhibit impaired motor performance and reduced open field rearing but normal elevated plus maze behavior. (**A**,**B**) Plotted are average values ± SEM for each 5 min. interval of a 60 min, initial exposure to a novel open field environment. Average of 17 RXRγ KO animals is shown in gray and 17 wild-type siblings in black. (**A**) No significant differences were observed between these groups for: time spent in the center (2-way RM-ANOVA: F(1,32) = 1.862, P = 0.1861 for genotype effect), or total time in the center (WT: 1022 ± 78 s; KO: 822.6 ± 125.0, T-test: t = 1.351, P = 0.1861). (**B**) RXRγ KO mice did exhibit a significant reduction in time spent rearing across blocks (2-way RM-ANOVA: F(1,32) = 8.131, P = 0.0076) as well as total time spent rearing (WT: 61.28 ± 3.577 s; KO: 44.35 ± 4.738 s; T-test: t = 2.851, P = 0.0076). (**C**) Average time spent ± SEM in open arms, closed arms and center of an elevated plus maze during a 6 min exposure for 18 RXRγ KO animals (gray) and 18 wild-type siblings (black). No significant differences were observed between these groups in the ratio of time spent in open vs. closed arms (WT: 0.3939 ± 0.04553; KO: 0.4312 ± 0.05536; T-test: t = 0.5196 P = 0.6067). (**D**Average latency to fall during 3 trials per day on each of 4 training days of an accelerating rotarod task for 18 RXRγ KO animals (gray) and 18 wild type siblings (black). 2-way RM-ANOVA with genotype and training day as factors shows a significant effect of genotype (F(1,70) = 5.099, P = 0.0271). Data were plotted using Prism (https://www.graphpad.com/scientific-software/prism/) and the figure assembled using Affinity Designer (https://affinity.serif.com/en-gb/designer/) software.

**Figure 5:**
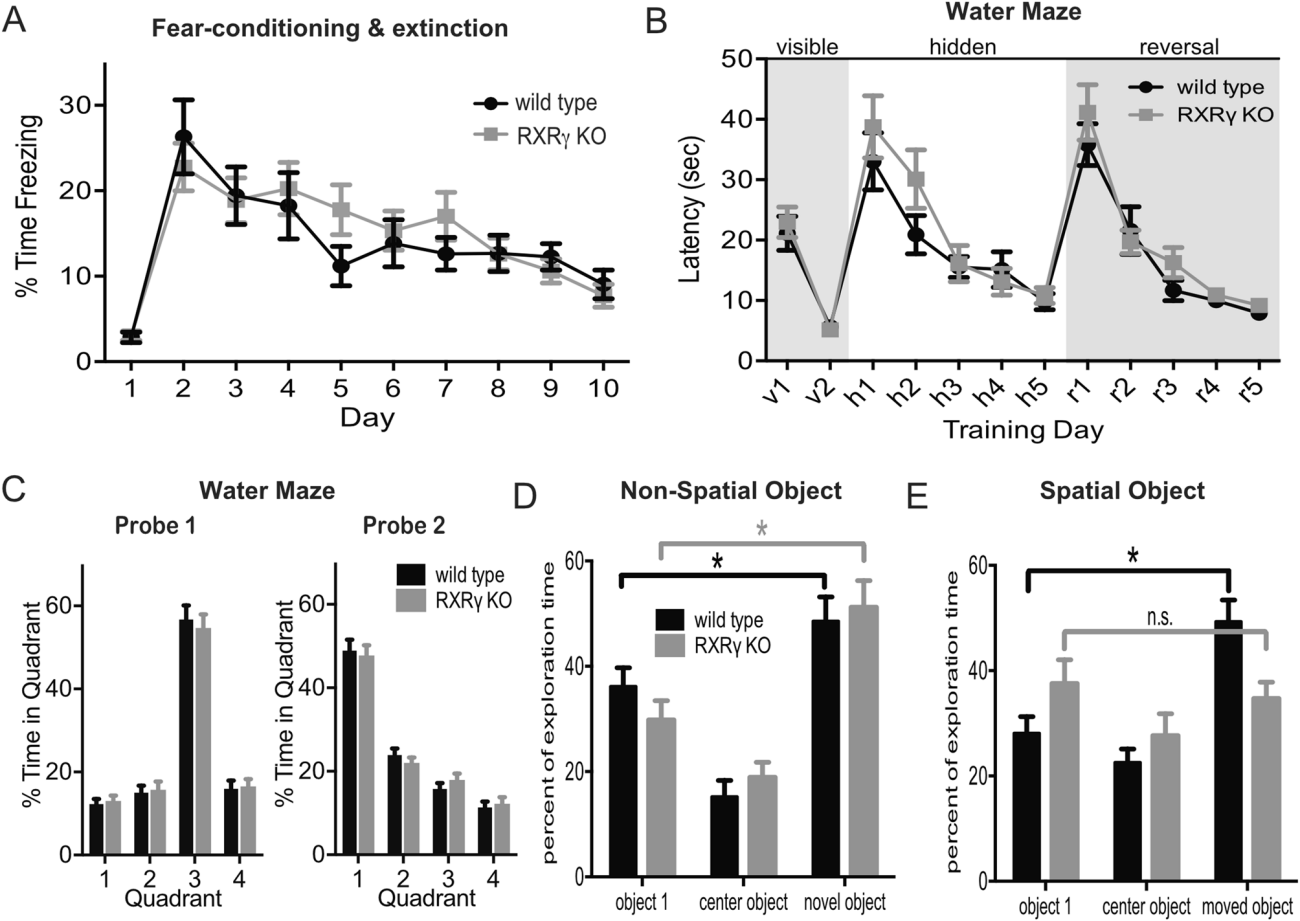
Animals lacking RXRγ exhibit impaired performance in a spatial version of a novel object recognition task, but normal performance in non-spatial object recognition, Morris water maze learning, and contextual fear conditioning. (**A**) Plot of average percent of time spent freezing ± SEM during 2 min of pre-shock exposure to a conditioning chamber as well as the first 2 min of each 10 min re-exposure to that environment on each of the following 9 days for 18 RXRγ KO animals and 16 wild type siblings revealed no differences in baseline freezing (T-test: t = 0.2340, P = 0.8165), conditioned fear response at 24 h (T-test: t = 0.7031, P = 0.4871), or extinction of conditioned fear response (2-way RM-ANOVA: F(8,256) = 21.97, P < 0.0001 for effect of trial, F(1,32) = 0.1310, P = 0.7198 for genotype). (**B**) Plot of average latency to reach escape platform ± SEM over 4 trials per day during the visible platform, hidden platform, and reversal phases of a Morris water maze task for 17 RXRγ KO animals and 17 wild type siblings revealed no differences between groups (2-way RM-ANOVA: F(1,32) = 0.1200, P = 0.7313, F(1,32) = 2.116, P = 0.1555, and F(1,32) = 1.195, P = 0.2825 for genotype during visible, hidden and reversal stages respectively). (**C**) Histograms of average percent time ± SEM spent in each of 4 quadrants of the water maze during 1 min probe trials conducted at the end of day H5 (probe 1) and R5 (probe 2) of the water maze task shown in B revealed no significant difference between RXRγ KO animals (gray) and wild type siblings (black) (2-way RM-ANOVA: F(3,96) = 128.9, P < 0.0001 for quadrant, and F(1,32) = 1.336, P = 0.2563 for genotype for probe 1; and F(3,96) = 123.5, P < 0.0001 for quadrant, and F(1,32) = 0.2819, P = 0.5991 for genotype for probe 2). (**D**) Histogram of average percent of time ± SEM spent exploring two familiar objects (object 1 and center object) and one novel object during a non-spatial novel object recognition test revealed no differences between RXRγ KO animals (N = 17) and wild type siblings (N = 20) (2-way ANOVA shows significant effect of object (F(2,70) = 23.77, P < 0.0001) but not genotype (F(1,35) = 1.094, P = 0.3028). Tukey’s post-hoc test reveals significant differences in exploration time for object 1 vs. novel object (P = 0.0051 for WT and 0.0088 for KO). (**E**) Histogram of average percent time ± SEM spent exploring two familiar objects (object 1 and center object) and one novel object during a spatial version of a novel object recognition test revealed significant differences between RXRγ KO animals (N = 12) and wild type siblings (N = 12). 2-way ANOVA shows significant object × genotype interaction (F(2,44) = 4.102, P = 0.0233). Tukey’s post-hoc test reveals significant differences in exploration time for object 1 vs. novel object for wild type (P = 0.0046), but not RXRγ KO animals (P = 0.8940). Data were plotted using Prism (https://www.graphpad.com/scientific-software/prism/) and the figure assembled using Affinity Designer (https://affinity.serif.com/en-gb/designer/) software.

Group 1 mGluR antagonists have been found to produce anxiolytic effects in animal models (reviewed in^24^). To assess effect of loss of RXRγ on anxiety, we compared the behavior of RXRγ knockout animals and wild type control siblings in a novel open field environment and in an elevated plus maze. We found that RXRγ knockout animals exhibited a slight reduction in the amount of time spent in the center of the open field that was not statistically significant compared to their wild type control siblings (Fig. 4A), and a significant reduction in the amount of time spent rearing (Fig. 4B). While these results are suggestive of a possible modest anxiogenic effect of the loss of RXRγ, we observed no difference between RXRγ knockout animals and wild type siblings in the amount of time spent in the open vs. closed arms of an elevated plus maze (Fig. 4C). Neither did these groups differ in the amount of time spent freezing during pre-shock exposure to a contextual fear conditioning apparatus (Fig. 5A). Both groups of animals travelled comparable distances during open field and elevated plus maze testing (Figs. S2A,C) and spent comparable amounts of time resting (immobile) in the open field (Fig. S2B), suggesting that their behavior in these tasks was not affected by differences in activity level. The behavior of RXRγ knockout animals we observe in the open field and elevated plus maze is consistent with previous studies on an independently generated line of RXRγ knockout mice^20,23^.

### RXRγ knockout impairs motor performance

Group 1 mGluRs play a role in normal motor function and learning by regulating neuronal activity in both cerebellar^43,44^ and basal ganglia circuits^45^, and mGluR1 (but not mGluR5^46^) knockout mice have been found to exhibit deficits in motor coordination^47–49^. A role for group 1 mGluRs has also been suggested in Parkinson’s disease and mGluR5 antagonists have shown promise for the treatment of levodopa-induced dyskinesia (reviewed in^24^). To assess the effect of RXRγ knockout on motor performance and learning, we tested these animals and their wild-type siblings on an accelerating rotarod task. We found that loss of RXRγ resulted in a significant decrease in motor performance in this task (Fig. 4D) that was consistent with the trend observed previously for RXRγ knockout animals on a continuous speed, single day version of this task^23^. These data are consistent with the possibility that impaired group 1 mGluR signaling in the RXRγ knockout mice may contribute to reduced motor performance in these animals.

### RXRγ knockout impairs spatial novel object recognition

Group 1 mGluR signaling is involved in changes in synaptic efficacy that are thought to be required for normal hippocampus-dependent learning and memory, and genetic or pharmacological interference with group 1 mGluRs has been found to cause deficits in spatial learning and memory (reviewed in^50^). To determine whether RXRγ might also be required for normal spatial learning and memory, we compared the performance of RXRγ knockout animals to that of their wild type siblings in a contextual fear conditioning task. Both mGluR1 and mGluR5 knockout animals have been found to exhibit deficits in this task^47,51,52^. However, the freezing response of RXRγ knockout animals was comparable to controls both during initial exposure to a novel context, and during re-exposure to that context 24 h after CS-US pairing (Fig. 5A). Loss of mGluR5 has also been suggested to interfere with the extinction of contextual fear conditioning^52^, however, RXRγ knockout animals exhibited a normal decline of the conditioned fear response during repeated exposure to the context in the absence of foot shock (Fig. 5A). Together these data suggest that loss of RXRγ does not alter contextual fear conditioning or extinction of the conditioned fear response.

As a second test for the possible effects of loss of RXRγ on hippocampus-dependent learning and memory, we compared the performance of RXRγ knockout animals to that of their wild-type siblings during visible platform, hidden platform, and reversal phases of a Morris water maze task. Both RXRγ knockout and wild type siblings rapidly learned the visible version of the water maze, suggesting that no task-relevant differences in swimming ability, visual acuity, or motivation exist between these groups (Fig. 5B, days v1–2). Swimming speed was also comparable between these two groups during all stages of water maze testing (Fig. S3A). Neither were significant differences observed between mutants and controls in the acquisition of a hidden platform version of this task (Fig. 5B, days h1–5) or in performance during a probe trial conducted at the end of hidden platform training (day h5) (Fig. 5C, probe 1). This result is in agreement with previously reported data^18^, and despite the observation that mice lacking mGluR5 show significant impairments in this task^51,52^. mGluR5 knockout mice have also been reported to exhibit a deficit in the reversal phase of this task^52^—wherein they are required to navigate to the platform in a new location. However, we observed no significant differences between RXRγ knockout animals and their wild type siblings during reversal training (Fig. 5B, days r1-5) or in performance during probe trial conducted on the last day of reversal training (day r5) (Fig. 5C, probe 2).

Animals lacking RXRγ were previously reported to exhibit deficits in novel object recognition^22^. Two versions of this task exist—one that requires hippocampus-dependent spatial memory in which animals recognize familiar objects in novel locations^53^, and a second, non-spatial version, that requires group 1 mGluR-mediated LTD in the perirhinal cortex in which animals recognize novel objects in familiar locations^54^. Since a previous report of novel object recognition impairments in RXRγ knockout animals did not distinguish between these two modalities, we sought to determine which form(s) of novel object recognition might be affected by loss of RXRγ. We found that RXRγ knockout animals and wild-type siblings showed similar preferences for novel versus familiar objects in the non-spatial version of this task (Fig. 5D), suggesting that loss of RXRγ did not affect the recognition of novel objects in familiar locations. However, we did observe a significant difference in the performance of these groups in the spatial version of the novel object recognition task. In this version, RXRγ knockout animals failed to distinguish familiar objects in novel locations under conditions where their wild-type siblings showed a clear preference (Fig. 5E), suggesting that RXRγ is required for the spatial but not the non-spatial form novel object recognition. In neither version of the task did the RXRγ knockout affect total exploration time (Fig. S3B,C). The observation that loss of RXRγ impairs performance in a spatial novel object recognition, but not contextual fear conditioning or Morris water maze tasks, suggests that the novel object recognition impairment may be related to the reported association between mGluR-dependent LTD and the response to novel environments and stimuli^50^.

### RXRγ knockout mice show reduced pre-pulse inhibition and working memory performance

Group 1 mGluR signaling has also been implicated in the pathology of schizophrenia (reviewed in^24^). In support of this hypothesis, mutations in mGluR1 and mGluR5^55–59^, and changes in mGluR1 expression^60–62^ are associated with schizophrenia in human patients, and genetic and pharmacological inhibition of group 1 mGluR signaling results in schizophrenia-related impairments in rodents both alone and in combination with NMDAR antagonist administration (reviewed in^63,64^). While potentiation of NMDAR function was thought to underlie the salutary effects of group 1 mGluR positive allosteric modulators (PAMs) on schizophrenia-related phenotypes, recent evidence suggests that group 1 mGluR PAMs may exert these effects via NMDAR-independent mechanisms as well^65^. A possible role for retinoid signaling in schizophrenia pathogenesis and treatment has also been suggested^17^, and mice lacking RXRγ have been reported to exhibit deficits in working memory that are also a characteristic schizophrenia-associated cognitive impairment^21,22^. Our observation that loss of RXRγ impairs group 1 mGluR responses suggests that reduced group 1 mGluR activity may underlie, or contribute to schizophrenia-related impairments in RXRγ knockout mice.

**Figure 6:**
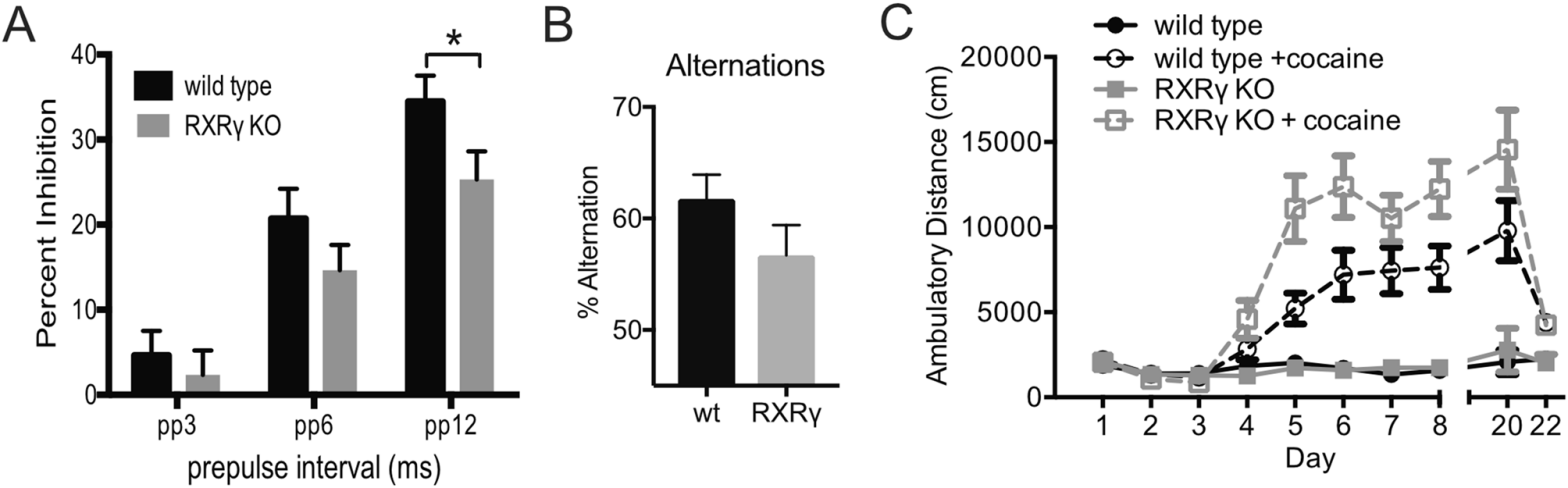
Animals lacking RXRγ exhibit reduced pre-pulse inhibition and increased sensitization to repeated cocaine exposure. (**A**) Histogram of the average percent of pre-pulse inhibition ± SEM at three different prepulse amplitudes (3, 6 or 12 dB above background, pp3, pp6 and pp12 respectively) revealed reduced prepulse inhibition in RXRγ KO animals that was statistically significant at the highest prepulse amplitude tested (T-test: t = 2.036, P = 0.0482). (N = 22 RXRγ KO and 21 wild type siblings). (**B**) Histogram of the average percent of spontaneous alternations during testing in a Y-maze revealed a trend for reduced alternation in RXRγ KO animals compared to their wild type siblings that was not statistically significant (T-test: t = 1.460 P = 0.1508). (RXRγ KO N = 19, WT N = 30). (**C**) Plot of average distance travelled ± SEM during a 30 min open field exposure following repeated administration of cocaine or saline showed significantly enhanced locomotor sensitization in RXRγ KO animals compared to wild type siblings. 2-way RM-ANOVA over days 5–20 shows a significant effect of genotype (F(3,49) = 21.29, P < 0.0001), and Dunnett’s multiple comparisons revealed significant differences between cocaine treated RXRγ animals and all other groups, (vs. wt + vehicle: P < 0.0001, vs. wt + cocaine: P = 0.0073; vs. RXRγ + vehicle: P < 0.0001). Group sizes were N = 12 for KO + saline, N = 15 for KO + cocaine, N = 12 for WT + saline, N = 14 for WT + cocaine. Data were plotted using Prism (https://www.graphpad.com/scientific-software/prism/) and the figure assembled using Affinity Designer (https://affinity.serif.com/en-gb/designer/) software.

To pursue this association further, we compared pre-pulse inhibition in RXRγ knockout mice and their wild type siblings. Prepulse inhibition is a behavioral paradigm designed to assess sensory-motor gaiting that has been found to be reduced in schizophrenic patients (reviewed in^66,67^) as well as mGluR1 knockout, mGluR5 knockout, and NMDAR antagonist-treated mice^68–73^. We found reduced prepulse inhibition in RXRγ knockout mice across a range prepulse amplitudes that reached statistical significance at the highest amplitude tested (Fig. 6A). The RXRγ knockout mutation was without effect on either the amplitude of the startle response (Fig. S4A), or the startle response time (Fig. S4B), suggesting that the reduced prepulse inhibition we observed in these animals is the result of an impairment in sensory-motor gaiting.

Impairments in working memory are also considered an endophenotype of schizophrenia, and have been described previously in mice lacking RXRγ^21,22^. To verify this impairment, we tested RXRγ knockout animals in a Y-maze spontaneous alternation task as described in^22^ and found a similar trend for reduced spontaneous alternation (Fig. 6B) that was consistent with, but lower in magnitude than previously reported.

### RXRγ knockout mice show increased locomotor sensitization to cocaine

Group 1 mGluRs are involved at multiple levels in the synaptic changes that are thought to underlie the behavioral response to drugs of abuse^31,74^, and a majority of data suggest that inhibition of mGluR1/5 signaling may alleviate symptoms associated with addiction (reviewed in^24^). To examine the effect of reduced RXRγ activity on addiction-related behavioral responses, we compared the locomotor activity of RXRγ knockout and wild-type animals that received daily doses of 15 mg/kg cocaine. Surprisingly, we found significantly higher cocaine-induced locomotor sensitization in RXRγ knockout animals compared to their similarly treated wild-type siblings (Fig. 6C). This is in contrast to the reduced amphetamine-induced locomotor responses described in mice treated with the RXR antagonist HX531^75^ and the reduced locomotor activity observed in RARβ/RXRγ and RXRβ/RXRγ double knockout mice that received a single cocaine injection^23^. The increased cocaine-induced locomotor sensitization we observe in RXRγ knockout animals may reflect the of loss of RXRγ during development, the selective interference with a subset of addiction-related group1 mGluR signaling functions, or mGluR-independent effects on the mechanisms underlying locomotor sensitization. However, it is also possible that this response is a consequence of altered drug metabolism stemming from the role that rexinoid signaling plays in regulating cytochrome expression in the liver^76,77^.

### RXRγ knockout blocks DHPG-induced LTD, but does not rescue increased locomotor activity in *Fmr1* mutant mice

Group 1 mGluR activation causes an increase in the local translation of a subset of mRNAs in dendritic spines, leading to an increase in proteins that mediate group 1 mGluR-dependent LTD^78^. The activating effect of group 1 mGluRs on translation is opposed by the repressive effect that the fragile X mental retardation protein (FMRP) exerts on these mRNAs, and accordingly, an increase in group 1 mGluR-dependent LTD is observed in Fmr1 mutant mice that lack FMRP^79^. This antagonistic relationship between the actions of group 1 mGluRs and FMRP on mRNA translation led to the proposal that inhibiting group 1 mGluR signaling might counter the effect of reduced FMRP function in patients with fragile X syndrome, and ameliorate some of the associated symptoms^80^. While this strategy has thus far proved ineffective in treating fragile X syndrome patients in clinical trials, multiple studies have reported that reducing group 1 mGluR signaling does effectively reverse fragile X syndrome-related phenotypes in mouse models of the disease^81,82^.

**Figure 7:**
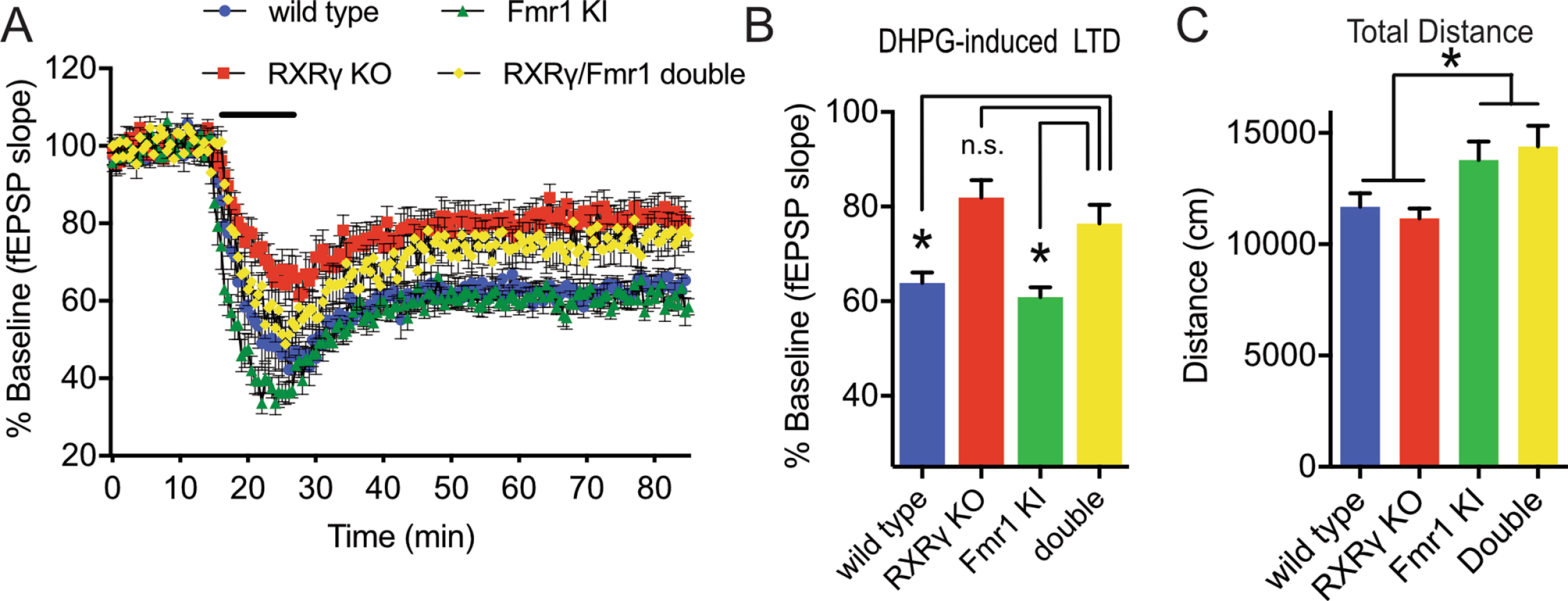
RXRγ knockout blocks DHPG-induced LTD, but not increased locomotor activity in Fmr1 mutant mice. (**A**) Time course of mean initial slopes ± SEM of fEPSPs at Schaffer collateral-CA1 synapses in slices from wild-type (N = 12 slices from 5 animals), RXRγ KO (N = 15 slices from 10 animals), hemizygous Fmr1 mutant (N = 14 slices from 4 animals), and RXRγ/Fmr1 double mutant mice (N = 11 slices from 3 animals), before, during, and after 10 min treatment with 100 μM DHPG (black bar). (**B**) Histogram of mean ± SEM evoked responses over the last 10 min of the recordings shown in (**D**). 2-way ANOVA with RXRγ and Fmr1 genotypes as factors revealed a significant effect of RXRγ genotype (F (3,40) = 252.6, P < 0.0001). Dunnett’s multiple comparison test showed significant differences between RXRγ/Fmr1 double mutant and both the wild-type (P = 0.0009) and Fmr1 mutant groups (P = 0.0061), but not the RXRγ KO group (P = 0.6019), indicating that loss of RXRγ impaired LTD in both Fmr1 mutant and wild-type animals. (**C**) Histogram of total distance traveled ± SEM during a 55 min exposure to a novel open field environment for 19 wild-type, 19 RXRγ KO, 20 Fmr1 mutant, and 17 RXRγ/Fmr1 double mutant animals showed that loss of RXRγ failed to rescue hyperactivity caused by the Fmr1 mutation (2-way ANOVA shows a significant effect of Fmr1 genotype, F(1,71) = 13.51, P = 0.0005, but not RXRγ genotype, F(1,71) = 0.0049, P = 0.9443). Data were plotted using Prism (https://www.graphpad.com/scientific-software/prism/) and the figure assembled using Affinity Designer (https://affinity.serif.com/en-gb/designer/) software.

To explore the possibility that RXRγ-dependent reductions in group 1 mGluR signaling might affect fragile X syndrome-related phenotypes in a mouse model of fragile X, we crossed RXRγ knockout mice with Fmr1^I304N^ mutant mice that carry a disease-linked point mutation in the gene encoding FMRP^83^. In wild-type hippocampal slices, DHPG treatment elicits a form of LTD that depends on the rapid induction of local protein synthesis, however, in Fmr1^I304N^ mutant mice, DHPG-induced LTD is no longer protein synthesis-dependent^83^. As shown in Fig. 7A,B, we found that the RXRγ/Fmr1^I304N^ double mutant mice showed significantly less LTD than mice carrying the Fmr1^I304N^ mutation alone, consistent with a general RXRγ-dependent impairment in group 1 mGluR signaling. However, we failed to observe significantly enhanced DHPG-induced LTD under these conditions in mice carrying the Fmr1^I304N^ mutation alone. To determine whether loss of RXRγ might also affect the behavioral phenotypes of mice that carry the Fmr1^I304N^ mutation, we compared the open field locomotor activity among siblings with these same mutant combinations. As reported previously^83^, we found that hemizygous Fmr1 knock-in mice exhibited increased locomotor activity compared to their wild-type siblings. However, unlike the effect of RXRγ knockout on DHPG-induced LTD, loss of RXRγ did not significantly impact the locomotor activity of RXRγ/Fmr1 double mutant animals when compared to siblings that carried the Fmr1 knock-in mutation alone (Fig. 7C). Together, these data are consistent with the notion that, while loss of RXRγ impairs group 1 mGluR signaling, the actions of group 1 mGluR signaling and FMRP do not overlap completely, and therefore, impairments in group 1 mGluR signaling caused by RXRγ knockout may affect only a subset of Fmr1 mutant phenotypes.

## Discussion

The electrophysiological data we present here suggest that RXRγ is required for normal group 1 mGluR-dependent electrophysiological responses in CA1 pyramidal neurons in the murine hippocampus. This conclusion is supported by impairments in mGluR agonist-induced LTD, mGluR agonist-induced calcium release, and mGluR agonist-induced activation of voltage-sensitive currents in RXRγ knockout mice. RXRγ knock out also impaired group 1 mGluR dependent LTD induced by paired-pulse stimulation in the presence of an NMDA receptor antagonist. These LTD impairments that we observe in the RXRγ knockout mice are similar to the impairments described in mice carrying a knockout mutation in a downstream effector of group 1 mGluR signaling—Gα_q_^84^. Given that loss of RXRγ also impairs LTD induced by low-frequency stimulation (LFS), and that this form of LTD depends on NMDA receptor activation, it is perhaps surprising to find that RXRγ knockout did not affect LTD induced by direct pharmacological activation of NMDA receptors^35,36^. While the basis for the effect of RXRγ knockout on LFS-induced LTD remains unclear, it may reflect impaired mGluR modulation of NMDA receptor activity, impaired mGluR-mediated metaplasticity, or another mGluR-dependent or independent effect on this form of LTD^85–87^. Future experiments to distinguish among these possible mechanisms may yield additional insights into the interaction between RXRγ and mGluR-dependent regulation of synaptic plasticity.

Given the involvement of group 1 mGluRs in multiple disease-relevant processes, we sought to determine the extent to which RXRγ-dependent impairments of group 1 mGluR signaling might affect behaviors in which these receptors are implicated. Our behavioral analysis identified impairments in a subset of group 1 mGluR-linked behaviors in mice lacking RXRγ. These impairments included: (1) impaired motor performance—suggesting that RXRγ-dependent decreases in group 1 mGluR signaling may underlie or contribute to this phenotype, (2) impaired prepulse inhibition that is consistent with the dependence of this behavior on normal group 1 mGluR activity and the role of group 1 mGluRs in this and other schizophrenia-related behaviors^24^, and (3) impaired recognition of familiar objects in novel locations, but not novel objects themselves, that is consistent with the reported relationship between hippocampal LTD and the response of mice to novel environments^88–90^.

Despite these examples of correspondences between the effect of the RXRγ knockout mutation on mGluR-dependent electrophysiological responses, and its effects on mGluR-dependent behaviors, we also identified examples of apparent dissociations. For example, we found that loss of RXRγ did not alter anxiety or extinction, despite the well-established anxiolytic effects of mGluR antagonists^24^ and the evidence linking group 1 mGluR activity to extinction^52^. Loss of RXRγ also did not affect non-spatial novel object recognition learning, despite considerable evidence linking mGluR-dependent LTD in the perirhinal cortex to these behaviors^54^. While loss of RXRγ still impaired DHPG-induced LTD in Fmr1 mutant mice, loss of RXRγ had no detectable effect on increased locomotor activity in these animals. The differential effects of loss of RXRγ on mGluR-dependent behaviors suggest that loss of RXRγ may affect mGluR activity differently in different brain regions. For example, loss of RXRγ may affect hippocampus-dependent spatial novel object recognition, but not perirhinal-dependent non-spatial novel object recognition—or loss of RXRγ may modulate only a subset of the downstream effects of mGluR activation. These dissociations are perhaps to be expected given the number of downstream effectors of group 1 mGluRs, their wide distribution throughout the brain, and the multiple forms of synaptic plasticity in which group 1 mGluRs play a role^24,31,32^. An understanding of the basis for the differential effects of RXRγ on group 1 mGluR-dependent behaviors will require an examination of its modulation of additional mGluR-dependent electrophysiological effects in different brain regions.

It will also be informative to determine the molecular basis for the genetic interaction between RXRγ knockout and group 1 mGluR signaling. Our data suggest that RXRγ-dependent changes in group 1 mGluR expression are not the basis for this interaction, raising the possibility that RXRγ-dependent changes in the expression of one or more group 1 mGluR downstream effectors or interacting proteins may lead to the impairments in group 1 mGluR mediated responses we observe. It is also possible that RXRγ exerts its effects on group 1 mGluR signaling via mechanisms that do not involve RXRγ-dependent changes in gene expression, but involve instead as yet unidentified direct molecular interactions with downstream targets analogous to the non-genomic actions of other nuclear hormone receptors^9^. An understanding of the molecular basis for the interaction between RXRγ and group 1 mGluR signaling may also suggest a role for RXRγ in mediating or modulating the electrophysiological and behavioral actions of other G-protein coupled receptors.

Our finding that loss of RXRγ inhibits group 1 mGluR electrophysiological responses and affects a subset of group 1 mGluR-dependent behaviors raises the possibility that pharmacologically targeting RXRγ could represent a viable therapeutic avenue for the treatment of diseases in which alterations in group 1 mGluR signaling play a role. Obstacles to this approach include the complexity of RXR’s interactions with multiple nuclear hormone receptor family members, and the lack of compounds that specifically target individual RXR isoforms. Nevertheless, RXR’s have been targeted successfully in the treatment of cancer despite these obstacles^3,9^. In addition to the potential therapeutic implications of these results, our data point to an interaction between two signaling pathways with established roles in regulating neuronal physiology and synaptic plasticity that could function under normal physiological conditions to integrate the cellular response to coincident stimulation of these pathways. It is likely that similar points of interaction exist among other signaling pathways, and that their continued identification will ultimately transform our current view of the molecular mechanisms that regulate neuronal physiology into a complex web of interdependent molecular signaling interactions that offer new insights and targets for disease intervention.

## Methods

### Animals

RXRγ knockout animals were described previously^18^ and were generously provided by R. Evans (Salk Institute). Animals were received in a mixed background and independently backcrossed for more than 5 generations to either C57BL6/J or 129SVEV/TAC wild type animals to generate congenic lines. Fmr1^I304N^ mutant mice^83^ were obtained from the Jackson Laboratory (Bar Harbor, ME) in a C57BL6/J background and crossed to C57BL6/J congenic RXRγ mice to generate double heterozygous females in this background. All animals used for experiments were F1 hybrids of the indicated ages, generated by crossing heterozygous or double heterozygous C57BL6/J animals to 129SVEV/TAC RXRγ heterozygous animals. Animals were maintained under standard conditions in ventilated cages on a 12 h light–dark cycle and tested during the day. Wild-type and knockout siblings were housed together in groups of 3–5 and run on behavioral experiments in age-matched cohorts by experimenters blinded to genotype. All experiments were carried out in a manner consistent with NIH guidelines and approved by the Columbia University and New York Medical College Institutional Animal Care and Use Committees (IACUC), and described in accordance with the ARRIVE 2.0 guidelines^91^.

### Slice preparation

Acute hippocampal slices were prepared as described previously^92^. Wild-type and homozygous knockout mice of mixed sex, 3–6 months of age, were decapitated under deep isoflurane anesthesia, and the hippocampus plus entorhinal cortex dissected free from surrounding tissue and placed immediately in ice-cold artificial cerebrospinal fluid (ACSF) consisting of: 126 mM NaCl, 26 mM NaHCO_3_, 1.25 mM NaH_2_PO_4_, 5 mM KCl, 2 mM CaCl_2_, 2 mM MgCl_2_, and 10 mM D-glucose, continuously gassed with 95% O_2_/ 5% CO_2_ (pH 7.2–7.4). For extracellular field potential recordings, 400 μm-thick transverse slices were cut using a vibrating tissue slicer (VT1200S, Leica Biosystems, Wetzlar, Germany), transferred to an interface recording chamber, maintained at 33 °C and continuously perfused with ACSF (3 mL/min), where they were incubated for a minimum of 90 min prior to the start of recording. For whole-cell patch recordings from single CA1 pyramidal neurons, slices were incubated for 1–6 h in oxygenated ACSF at room temperature prior to transfer to a submerged recording chamber continuously perfused with room temperature ACSF (5 mL/min).

### Electrophysiological recordings

Extracellular population fEPSP recordings were made as described previously^93^ using glass microelectrodes (2–3 MΩ when filled with ACSF) placed in stratum radiatum of the CA1 region of hippocampal slices under visual guidance, to a depth of 100–150 μm. Bipolar stainless-steel stimulation electrodes (FHC, Bowdoin, ME) were placed in stratum radiatum to activate Schaffer collateral afferents. For baseline recordings, synaptic inputs were stimulated once per min (150 μs square DC pulse). Baseline stimulus strength (10–200 μA) was adjusted to elicit a response ≈ 50% of the maximum fEPSP amplitude prior to the generation of a population action potential, and monitored for at least 30 min prior to induction of LTD. Slices in which there was a drift in baseline of > 5% for the 6 points prior to stimulation or drug treatment were excluded from further analysis. NMDA receptor-dependent LTD was elicited with a 10 min train of 2 Hz low frequency stimuli^94^, while group I mGluR-dependent LTD was evoked in the presence of the NMDAR antagonist D-2-amino-5-phosphonopentanoic acid (D-AP5, 10 µM) by a 2 Hz/10 min train of *pairs* of stimuli (50 ms pair interstimulus interval). Synaptic strength was quantified by measuring the maximum slope of the initial falling phase of the fEPSP, using a six-point interpolation least-squares linear regression analysis, which marched along the response until the maximum value was retrieved. Signals were collected with an Axoclamp-2A amplifier (Molecular Devices, San Jose, CA) filtered at 1 kHz, sampled at 10 kHz, and digitized and analyzed on an PC computer using DataWave Technologies software (DataWave, Loveland, CO).

Whole-cell patch-clamp recordings (Axoclamp 700B, Molecular Devices) were obtained from CA1 pyramidal neurons visualized using infrared differential interference contrast optics on a Zeiss Axioskop FS upright microscope (Carl Zeiss, Thornwood, NY) as described previously^95^. The intracellular patch pipette filling solution contained: 130 mM CsMeSO_4_, 4 mM NaCl, 10 mM HEPES, 0.5 mM EGTA, 4 mM Mg-ATP, 0.3 mM Na-GTP, and 2 mM QX-314 (pH 7.25; 280–290 mOsm; 4–6 MΩ). EPSCs were acquired at 5 kHz sample frequency and filtered at 1 kHz with an eight-pole low-pass Bessel filter. After whole-cell configuration was established, membrane potential was voltage-clamped at − 60 mV. Recordings with leak currents of <  − 100 pA, series resistances > 25 MΩ, or series resistances that changed by > 10% from beginning to end of experiments, were discarded. Voltage-dependent inward currents were evoked by stepping to − 80 mV, and then injecting a 1 s current ramp from − 80 mV to + 40 mV.

### Calcium imaging

Calcium imaging was performed on proximal apical dendrites of CA1 pyramidal cells in acute hippocampal slice preparations as described previously^96^. A customized two-photon laser-scanning Olympus BX61WI microscope with a 60×/1.1 nA objective was used to detect Ca^2+^ signals. A Mai/Tai laser (Solid-State Laser, Mountain View, CA) tuned to 820 nm was used for excitation, and image acquisition and storage was controlled by Olympus Fluoview FV300 software (Olympus, Melville, NY). In the transfluorescence pathway, a 565 nm dichroic mirror was used to separate green and red fluorescence, passed through HQ525/50 and HQ605/50 emission filters, to eliminate transmitted or reflected excitation light (Chroma Technology, Rockingham, VT), and detected simultaneously by two photomultiplier tubes. Neurons were loaded with dyes through the patch pipette for 20 min before commencing image acquisition. Alexa Fluor-594 was used to outline neuronal dendritic structure, and Calcium Green-1 (100 µM in the patch pipette) to detect [Ca^2+^] changes. To measure [Ca^2+^] dynamics, fluorescence was collected by scanning at 4–5 Hz in a surface-scanning mode (XYT), or 1 kHz in XT mode, and averaged from specified structures to obtain F(t). Baseline fluorescence (F0) was calculated as the average of four images obtained immediately prior to bath application of 30 μM DHPG. Images of the same cells were obtained after 5, 10, 15 and 20 min of DHPG application. Normalized values for each cell at each time point were calculated by dividing the fluorescent signals at a given time point by the average fluorescent signal for that cell obtained prior to drug treatment (F0). No EGTA was added to the internal solution for [Ca^2+^] imaging.

### Western blots

Hippocampi were homogenized by sonication in 50 mM Tris pH 7.4/2% SDS followed by incubation at 95 °C for 5 min. Extracts were run in duplicate and resolved by denaturing PAGE and blotted to nylon membrane (Immobilon-FL, Millipore). Blocking and antibody incubations were for 1 h at room temperature in Tris-buffered saline, 0.05% tween 20, 3% non-fat dry milk. Washes were carried out TBS-Tween without milk. Proteins were detected using commercially available antibodies against mGluR1 (Millipore AB1551), mGluR5 (Millipore AB5675), and α-tubulin (Sigma T9026) in conjunction with dye conjugated anti-mouse or rabbit secondary antibodies (Li-COR, Lincoln, NE). Blots were scanned and integrated band intensities determined using an Odyssey infrared imaging system. mGluR1 or mGluR5 band intensities were normalized to the band intensity for tubulin in that lane as a loading control and data were presented as percent of the average normalized control value.

### Q-PCR

Total hippocampal RNA was prepared using Trizol Reagent and first strand cDNAs were prepared using the Superscript III first strand cDNA synthesis supermix for Q-PCR (Invitrogen) as per manufacturers instructions. Quantitect primer pairs were purchased from Qiagen. All primer pairs showed a linear response of Log(template dilution) vs. cycle threshold for template dilutions over 5 orders of magnitude. Optimal annealing temperatures, template dilutions and corresponding efficiencies were determined experimentally for each primer pair, and melting curves determined for each reaction showed a single peak under these conditions. Relative expression levels were calculated for each sample using the experimentally determined efficiency and the number of cycles to reach a florescence threshold set manually within the linear portion of a plot of the Log(florescence intensity) vs. cycle number. Values were normalized to those obtained using a primer pair specific for GAPDH run in parallel for each sample.

### Immunohistochemistry

Brains were fixed by transcardial perfusion in 4%PFA dissolved in 100 mM phosphate buffer pH 7.4. 30 μM sections were cut using a Vibratome slicer. Floating sections were permeablized for 30 min at 4 °C in Tris-buffered saline + 0.2% triton and blocked for 1 h at room temp in Tris-buffered saline + 10% fetal bovine serum. Primary antibody incubations were carried out overnight at 4 °C in blocking buffer, secondary antibody incubations were carried out for 1 h at room temperature also in blocking buffer, and washes were TBS + 0.2% triton. Primary antibodies were from Millipore as above, and an Alexa-568 anti-rabbit from Invitrogen was used as a secondary. Stained sections were mounted in Fluorsave aqueous mounting medium (Calbiochem) and images were obtained by confocal microscopy (Olympus Fluoview FV1000 and associated software).

### Open field

Behavior in a novel open field was assessed using Plexiglas activity chambers (model ENV- 520; Med Associates, St. Albans, Vermont) (43.2 cm long × 43.2 cm wide × 30.5 cm high). Mice were placed in the open field and activity was recorded for 60 min. Behavioral measures were calculated using the Activity Monitor program (Med Associates) and “center” was defined as an area beginning 10 cm from the walls.

### Elevated plus maze

Animals were placed on an elevated plus maze apparatus for 6 min and their position was tracked using an overhead digital video camera and Anymaze software (Stoelting, Wood Dale, IL). The apparatus consisted of a plus shaped track with arms 18 cm long and 5 cm wide. Two arms were enclosed on 3 sides and the entire apparatus was elevated 50 cm above the bench top and surrounded by a white curtain under ambient room lighting.

### Accelerating rotarod

Mice were tested on an accelerating rotarod apparatus (Letica LE8200) over a period of 5 days. The first day consisted of 3 pre-training trials in which animals were habituated to the stationary apparatus 2 times for 3 min each, and then for an additional 3 min at 4 rpm. Animals that fell from the apparatus during these trials were returned to the rod until 3 min had elapsed. On subsequent days, animals were given 3 × 5 min trials per day with the rotation speed ramped from 4 to 20 rpm over the course of the trial. Animals that fell from the rod during these trials were returned to the rod once to discourage them from learning to fall as an escape strategy, however only the latency to the first fall was used for analysis. For all trails on all days, the intertrial interval was approximately 30 min during which animals were returned to their home cages.

### Fear conditioning

All fear conditioning trials were carried out in conditioning chambers placed in sound attenuating chambers (Med Associates, St Albans, VT) and freezing during each trial was monitored continuously using a video tracking and analysis system (Freezeframe, Actimetrix Software, Wilmette, IL). On day one animals were given a 3 min training trial consisting of 2 min of pre-exposure followed by a 2 s 0.6 mA continuous foot shock and 58 s of post-shock exposure. On subsequent days animals were reintroduced to the conditioning chamber in the absence of shock for 10 min. Percent of time spent freezing during the first 2 min of these trails was used to monitor extinction of the freezing response.

### Morris water maze

The experiment was carried out in a circular pool 120 cm in diameter filled with water made opaque with white paint. A video tracking system (HVS Image VP-118) was used to record and analyze each animal’s behavior. A 10 cm square platform submerged 1 cm below the water surface was used as an escape platform and in the visible version of the task, a colored syringe barrel was affixed to the center to mark its location. For all phases of the experiment, 4 trials of no more than 120 s were given each day at 20 min inter-trial intervals. Animals that exceeded the 120 s limit were guided to the platform before being returned to their home cage. A single “shaping” trial in which animals were placed on the platform for 15 s, was performed before training on the first day of the visible, hidden and reversal phases of the task. Probe trials of 60 s, during which the platform was removed from the water maze, were performed at the end of training on days H5 and R5. In the visible platform phase of the task, the pool was surrounded by a curtain and the marked platform was placed in a different location on all four trials of a day. In the hidden and reversal phases, the curtain was removed to reveal the room cues and the hidden platform was placed in a fixed location for each phase.

### Spatial and non-spatial object recognition

For all object recognition experiments, the apparatus consisted of an opaque plexiglass arena 71 cm square and 36 cm high. The arena was surrounded by a white curtain, and two prominent visual cues were affixed to two of the arena walls. Objects were glass, metal or ceramic items fixed in place with double stick tape (eg. padlock, glass chess piece, metal bottle cap, ceramic drawer knob). All objects, the arena and floor were cleaned with non-alcoholic antiseptic wipes between each trial. To habituate the animals to the testing environment, animals were handled in the testing room on the three days prior to testing. Animal movements were tracked and analyzed using Noldus Ethovision XT software and an overhead-mounted video camera. For the novel object version of the task animals were placed in the empty arena for 5 min first. Then three 5 min training trails were conducted in which three different objects were placed equidistantly in a diagonal line across the arena. Finally a 5 min. testing trial was conducted in which one of two peripheral objects was replaced with a new object. The delay between the empty arena trial and all of the training trials were 10 min, and the delay between the final training trial and the testing trial was 3 h. The spatial version of this task was carried out similarly except that instead of replacing one of the peripheral objects in the testing trail, it was moved to one of the two empty corners so that the three objects were now arranged in a “V”.

### Prepulse inhibition

Testing was performed essentially as described in^97^ and^98^. Animals were placed in startle chambers (SR-Lab startle response system, San Diego Instruments, San Diego, CA) containing a high-frequency speaker for generation of acoustic stimuli and a piezoelectric accelerometer to measure animal movements in response to stimulus presentations. A continuous level of 65 dB background white noise was presented through the experiment. The testing protocol consisted of 6 presentations of a 40 ms broadband 120 dB startle tone, followed by 10 pseudo-randomly interleaved presentations of background alone, prepulse trials consisting of stimuli at 3, 6 or 12 dB above background delivered 100 ms before the 40 ms broadband 120 dB startle tone, and non-prepulse trials in which the 120 dB startle tone was presented alone. This protocol was followed by another 6 presentations of the 120 dB startle tone alone. The interval between trials varied pseudorandomly between 15 and 30 s. PPI for each animal at a particular prepulse interval was calculated as the difference between the average startle magnitude on interspersed startle tone-alone trials and prepulse trials divided by the average startle magnitude on the same startle tone-alone trials.

### Y-maze spontaneous alternation

Mice were tested individually for spontaneous alternation during a single 6 min trial in a custom fabricated Y-maze apparatus. The maze consisted of three arms 40 cm in length, 17 cm high and 4 cm wide, separated by 120 degrees located on a table in the center of the testing room under dim lighting. Animals were placed at the end of one of the arms in a psedorandomly assigned manner. Movements were recorded by an overhead mounted video camera and monitored by an observer blinded to genotype. Arm entries were scored when all four of the animal’s paws entered an arm. The percent alternation was calculated as the number of triads containing entries into all three arms divided by the total number of triads (the total number of arms entered minus 2) × 100. Average number entries for all groups was 24.2 with a standard deviation of 5.5. Three RXRγ knockout and 2 wild-type mice with 13 or fewer arm entries were excluded from the analysis.

### Behavioral sensitization to cocaine

Testing was conducted in the same apparatus used for open field behavior testing. Immediately before each 30 min trial, animals were given a single intraperitoneal injection of either 15 mg/kg cocaine or saline only. The testing protocol consisted of 3 days of habituation (1–3) in which all animals were administered saline only. Habituation was followed by 5 sensitization trials, conducted on days 4–8 and day 20, in which either cocaine or saline was administered as indicated. On day 22 all animals were tested again after receiving saline injections.

### Statistical analysis

Tests for statistical significance between groups were performed using Prism 7 (Graphpad Software, San Diego, CA, USA). Student’s unpaired, two-tailed t-tests were used for experiments involving 2 groups, and paired t-test for experiments in a single group comparing before to after drug application. 2-way ANOVA comparisons—with or without repeated measures as dictated by the experimental design—were used for analysis of all experiments involving multiple groups. Post-hoc comparisons for multiple group experiments were performed using Dunnett’s tests when comparisons were made to a single control group, and Tukey’s tests when comparisons were made among multiple groups. The results of these tests together with their associated P values are listed in the legends for their corresponding figure.

## Supplementary Information

Supplementary Information.
