# Supplementary material for: Loss of retinoid X receptor gamma subunit impairs group 1 mGluR mediated electrophysiological responses and group 1 mGluR dependent behaviors
Source: Sci Rep. 2021 Mar 10;11:5552. doi: 10.1038/s41598-021-84943-x (PMC7946894; doi:10.1038/s41598-021-84943-x)
Supplement: Supplementary file 1 — Supplementary Information. [file 41598_2021_84943_MOESM1_ESM.pdf]

TITLE

Loss of retinoid X receptor gamma subunit impairs group 1 mGluR mediated electrophysiological responses and group 1 mGluR dependent behaviors.

AUTHORS

Chirag Upreti[1], Caitlin M. Woodruff[3], Xiao-Lei Zhang[1], Michael J. Yim[3], Zhen-yu Zhou[1,2], Andrew M. Pagano[3], Dina S. Rehanian[4,5], Deqi Yin[3,6], Eric R. Kandel[3,6,7,8], Patric K. Stanton[1,2] Russell E. Nicholls[4,5],

Department of Cell Biology and Anatomy[1], Department of Neurology[2], New York Medical College, Valhalla, NY 10595, USA.

Department of Neuroscience[3], Howard Hughes Medical Institute[6], Kavli Institute for Brain Science[7], and Mortimer B. Zuckerman Mind Brain Behavior Institute[8], Columbia University, 3227 Broadway, New York, NY 10027, USA.

Department of Pathology and Cell Biology[4], Taub Institute for Research on Alzheimer's Disease and Aging Brain[5], Columbia University, 630 West 168th Street, New York, NY 10032, USA.

CORRESPONDING AUTHOR:

Russell E. Nicholls, Ph.D.
Department of Pathology and Cell Biology, and
The Taub Institute for Research on Alzheimer's Disease and the Aging Brain,
Columbia University
630 West 168th Street
New York, NY 10032, USA

Phone: 646-379-1812
Email: rn95@columbia.edu

Supplemental Material:

Figure S1: Sensitivity of LTD induced by 2Hz and 2 Hz paired-pulse stimulation to pharmacological inhibition of group 1 mGluR or NMDA receptors. A) Time courses of averaged initial fEPSP slopes ± SEM at Schaffer collateral-CA1 synapses in hippocampal slices from wild-type mice prior to and after 2 Hz/10 min low frequency stimulation (black bar) in the presence of ACSF containing 10 μM MCPG, 25 μM D-APV or vehicle (gray bar). ANOVA of mean responses relative to baseline over the last 10 minutes of recording showed a significant effect of group ($F(2,21) = 15.31$, $p < 0.0001$). Dunnett's pairwise comparisons show a significant difference between the APV and vehicle-treated groups ($p = 0.0002$), but not the MCPG and vehicle-treated groups ($p = 0.7363$). N= 10 vehicle, 8 APV, and 6 MCPG-treated slices. B) Time courses of averaged initial fEPSP slopes ± SEM at Schaffer collateral-CA1 synapses in hippocampal slices from wild-type mice prior to and after 2 Hz paired-pulse stimulation (black bar) containing 10 μM MCPG, 25 μM D-APV or vehicle (gray bar). ANOVA of mean responses relative to baseline over the last 10 minutes of recording showed a significant effect of group ($F(2,20) = 8.342$, $p = 0.0023$). Dunnett's pairwise comparisons show a significant difference between the MCPG and vehicle-treated groups ($p = 0.0011$), but not the APV and vehicle-treated groups ($p = 0.2118$). N= 9 vehicle, 8 APV, and 6 MCPG treated slices. Data were plotted using Prism (https://www.graphpad.com/scientific-software/prism/) and the figure assembled using Affinity Designer (https://affinity.serif.com/en-gb/designer/) software.

Figure S2: Representative signal-averaged traces before and after 2 Hz and DHPG-induced LTD in wild type and RXRγ KO mice. A) Representative traces of fEPSP responses at baseline (black traces, average of -5 to 0 min before LFS) and average of 55-60 min after LTD (gray traces) elicited by 1200 action potentials at a frequency of 2HZ (LFS) in wild-type and RXRγ KO slices from an experiment shown in Figure 1A. B) Representative traces of fEPSP responses at baseline (black traces, average of -5 to 0 min before LFS) and 55-60 min after LTD induction by 10 min bath application of DHPG (gray traces) by bath application of 30μM DHPG in wild-type and RXRγ KO slices from experiment shown in Figure 1B. All traces are average of 10 consecutive sweeps. Data were plotted using Microsoft Excel 16.44 and the figure assembled using Affinity Designer (https://affinity.serif.com/en-gb/designer/) software.

Figure S3: Uncropped images of representative western blots shown in Fig. 3. A) Anti-mGluR1 immunoreactivity at the expected molecular weight as indicated by arrow. B) Corresponding anti-tubulin immunoreactivity detected on a separate channel for the blot shown in A. C) Anti-mGluR5 immunoreactivity at the expected molecular weight as indicated by arrow. D) Corresponding anti-tubulin immunoreactivity detected on a separate channel for the blot shown in C. Images were prepared using Image Studio (https://www.licor.com/bio/image-studio/), and the figure was assembled using Affinity Designer (https://affinity.serif.com/en-gb/designer/) software.

Figure S4: Animals lacking RXRγ exhibit normal ambulatory activity in an open field and elevated plus maze. A-B) Plotted are average values ± SEM for each 5 min interval of a 60 min, initial exposure to a novel open field environment. Average of 17 RXRγ KO animals is shown in gray and 17 wild-type siblings in black. No significant differences were observed in: A) ambulatory distance traveled (RM-ANOVA: $F(1,32)= 3.17$, $P= 0.0845$) or B) resting time (RM-ANOVA: $F(1,32)= 2.564$, $P= 0.1191$). C) Average distance traveled ± SEM during a 6 min of testing in an elevated plus maze for 18 RXRγ KO animals (gray) and 18 wild-type siblings (black) revealed no significant differences between groups (WT: 1507 cm ± 43.95; KO: 1597 cm ± 46.90; T-test: $t= 1.398$, $P= 0.1713$). Data were plotted using Prism (https://www.graphpad.com/scientific-software/prism/) and the figure assembled using Affinity Designer (https://affinity.serif.com/en-gb/designer/) software.

Figure S5: Animals lacking RXRγ exhibit normal swim speed and object exploration. A) Plot of average swimming speed ± SEM over 4 trials per day during the visible platform (v1, v2), hidden platform (h1-5), and reversal phases (r1-5) of a Morris water maze task for 17 RXRγ KO animals and 17 wild-type siblings revealed no differences between groups (2-way RM-ANOVA for genotype: $F(1,64)= 0.1389$, $P= 0.7106$ for visible; $F(1,160)= 0.1274$, $P= 0.7216$ for hidden; $F(1,160)= 3.062$, $P= 0.0821$ for reversal). B) Plot of average time spent exploring all objects ± SEM during each 5 min trial of a non-spatial novel object recognition task shown in Fig 4D for 17 RXRγ KO animals and 20 wild-type siblings revealed no differences between groups (2-way RM-ANOVA, $F(1,35)= 0.2984$, $P =0.5884$ for genotype). Value for exploration time during empty arena test was generated by measuring the amount of time the animal's head was located

in the zones occupied by objects in subsequent trials. C) Plot of average time spent exploring all objects ± SEM during each 5 min trial of a spatial version of a novel object recognition task shown in Fig 4E for 12 RXRγ KO animals and 12 wild-type siblings revealed no differences between groups (2-way RM-ANOVA, $F(1,22)= 0.3397$, $P =0.5659$ for genotype). Value for exploration time during empty arena test was generated by measuring the amount of time the animal's head was located in the zones occupied by objects in subsequent trials. Data were plotted using Prism (https://www.graphpad.com/scientific-software/prism/) and the figure assembled using Affinity Designer (https://affinity.serif.com/en-gb/designer/) software.

Figure S6: Animals lacking RXRγ exhibit normal startle responses, and normal activity in the Y-maze apparatus. A) Histogram of average startle amplitude ± SEM for 22 RXRγ KO and 21 wild-type siblings in response to 10 interspersed, post-habituation presentations of the 120 dB startle tone alone showed no significant difference between groups (T-test: $t= 1.456$, $P =0.1530$). B) Plot of average startle response time ± SEM for 22 RXRγ KO and 21 wild-type siblings revealed no significant differences between genotypes ($F(1,41)= 0.3912$, $P =0.5351$ for 2-way RM-ANOVA with genotype and prepulse amplitiude as factors) in any of 3 types of prepulse inhibition trials: prepulse at 3 dB (pp3), 6 dB (pp6), or 12 dB (pp12) above background, or following presentation of the 120 dB startle tone alone (p120) (Fisher's LSD test KO vs. wt comparisons $P =0.6460$, 0.1662, 0.5265, and 0.8501 respectively). C, D) Histograms of the average distance traveled and average total arm entries during testing in a Y-maze revealed no significant differences between RXRγ KO animals and their wild-type siblings (T-tests: $t= 0.6262$, $P = 0.9945$ for distance, $t= 0.0069$, $P = 0.5342$ for arm entries). ($N = 19$ RXRγ KO and 30 wild-type siblings). Data were plotted using Prism (https://www.graphpad.com/scientific-software/prism/) and the figure assembled using Affinity Designer (https://affinity.serif.com/en-gb/designer/) software.

A

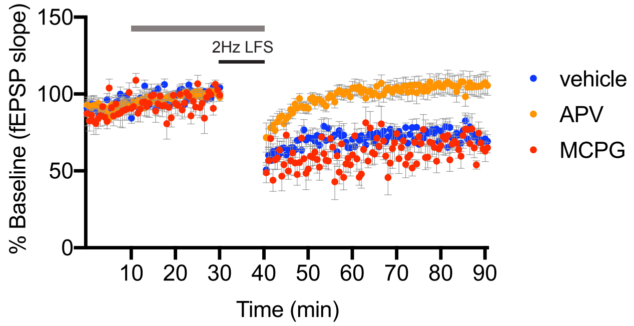

% Baseline (fEPSP slope)
150
100
50
0
2Hz LFS
10 20 30 40 50 60 70 80 90
Time (min)
vehicle
APV
MCPG

B

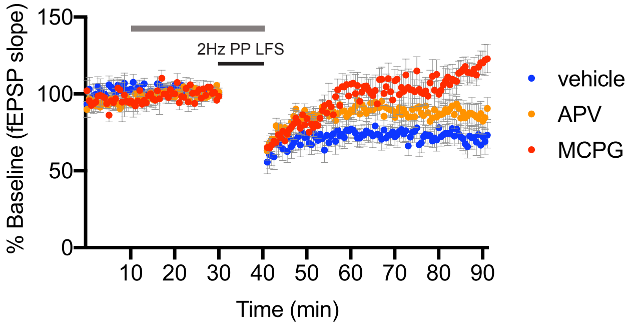

% Baseline (fEPSP slope)
150
100
50
0
2Hz PP LFS
10 20 30 40 50 60 70 80 90
Time (min)
vehicle
APV
MCPG

Fig S1

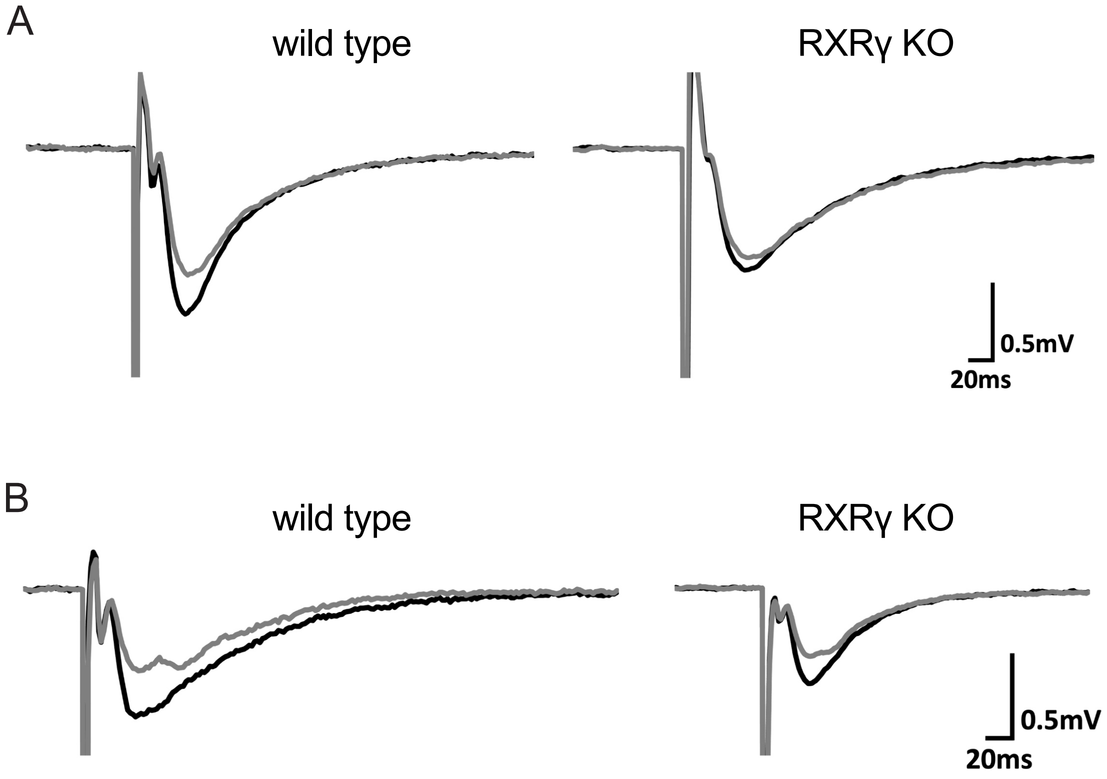

Fig S2

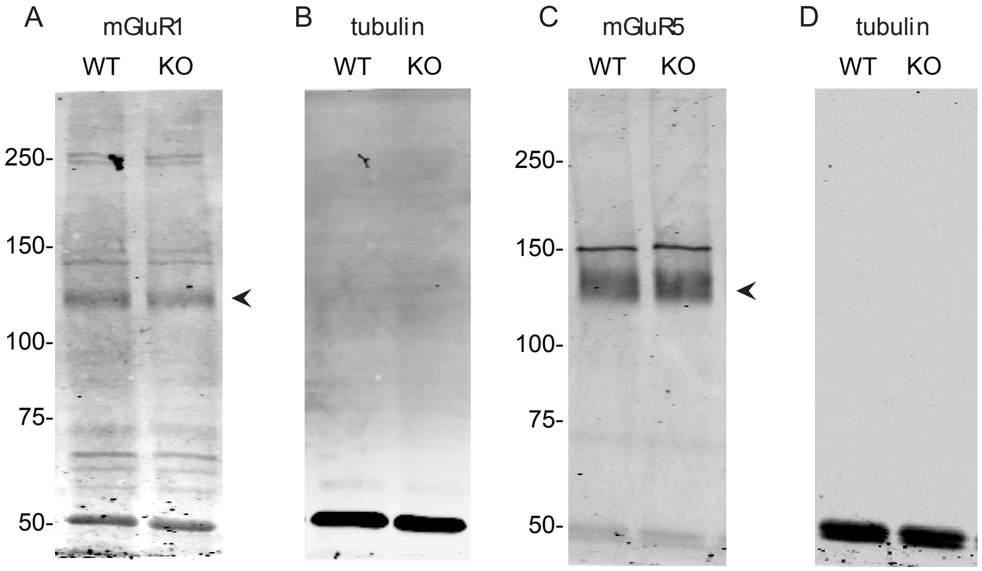
A
mGluR1
WT KO
250-
150-
100-
75-
50-
B
tubulin
WT KO
C
mGluR5
WT KO
250-
150-
100-
75-
50-
D
tubulin
WT KO

Fig S3

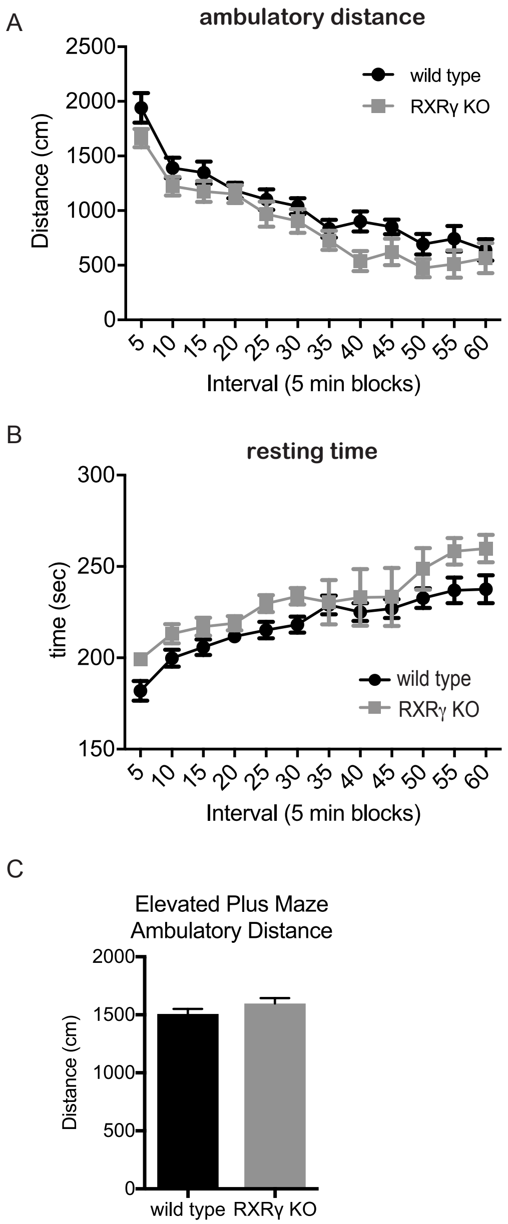

A
ambulatory distance
Distance (cm)
2500
2000
1500
1000
500
0
wild type
RXRγ KO
5 10 15 20 25 30 35 40 45 50 55 60
Interval (5 min blocks)
B
resting time
time (sec)
300
250
200
150
wild type
RXRγ KO
5 10 15 20 25 30 35 40 45 50 55 60
Interval (5 min blocks)
C
Elevated Plus Maze
Ambulatory Distance
Distance (cm)
2000
1500
1000
500
0
wild type
RXRγ KO

Fig S4

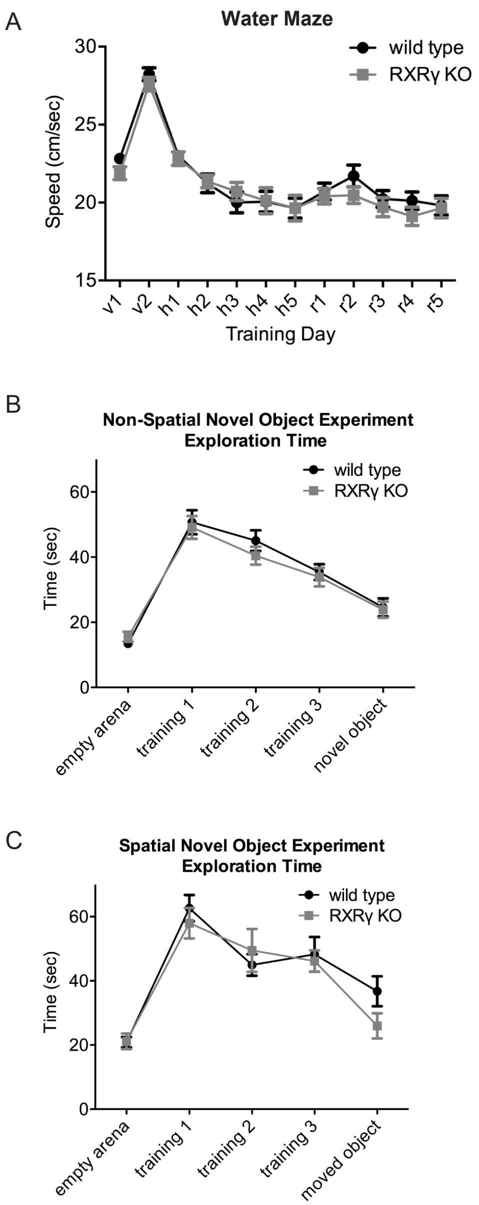
A
Water Maze
wild type
RXRγ KO
Speed (cm/sec)
30
25
20
15
v1
v2
h1
h2
h3
h4
h5
r1
r2
r3
r4
r5
Training Day
B
Non-Spatial Novel Object Experiment
Exploration Time
wild type
RXRγ KO
Time (sec)
60
40
20
0
empty arena
training 1
training 2
training 3
novel object
C
Spatial Novel Object Experiment
Exploration Time
wild type
RXRγ KO
Time (sec)
60
40
20
0
empty arena
training 1
training 2
training 3
moved object

Fig S5

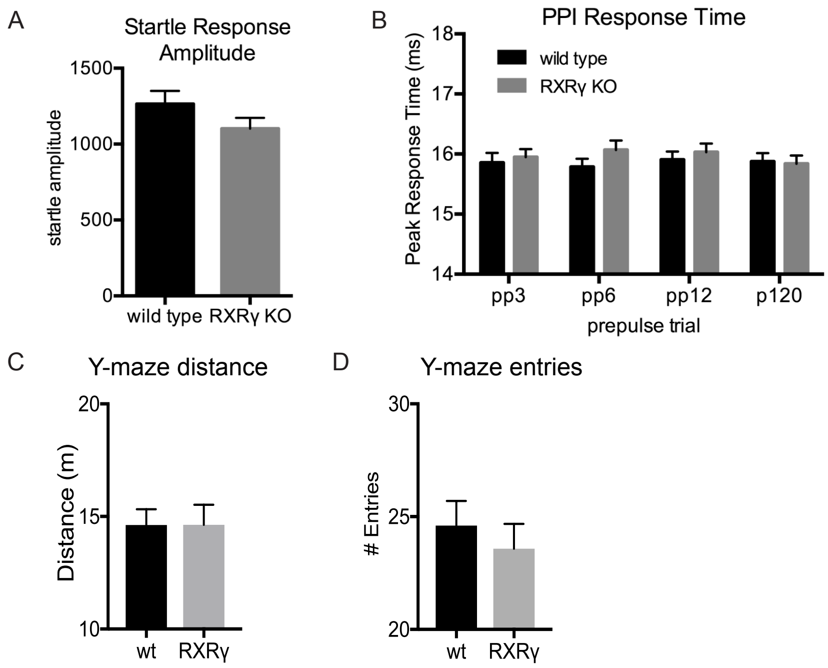
A
Startle Response Amplitude
startle amplitude
0
500
1000
1500
wild type
RXRγ KO
B
PPI Response Time
Peak Response Time (ms)
14
15
16
17
18
wild type
RXRγ KO
pp3
pp6
pp12
p120
prepulse trial
C
Y-maze distance
Distance (m)
10
15
20
wt
RXRγ
D
Y-maze entries
Entries
20
25
30
wt
RXRγ

Fig S6
